# Blood–brain barrier dysfunction in l-ornithine induced acute pancreatitis in rats and the direct effect of l-ornithine on cultured brain endothelial cells

**DOI:** 10.1186/s12987-022-00308-0

**Published:** 2022-02-17

**Authors:** Fruzsina R. Walter, András Harazin, Andrea E. Tóth, Szilvia Veszelka, Ana R. Santa-Maria, Lilla Barna, András Kincses, György Biczó, Zsolt Balla, Balázs Kui, József Maléth, László Cervenak, Vilmos Tubak, Ágnes Kittel, Zoltán Rakonczay, Mária A. Deli

**Affiliations:** 1 Institute of Biophysics, Biological Research Centre, Temesvári krt. 62, Szeged, 6726 Hungary; 2 Department of Medicine, University of Szeged, Kálvária sgt 57, Szeged, 6725 Hungary; 3 Department of Pathophysiology, University of Szeged, Semmelweis u. 1, Szeged, 6701 Hungary; 4 HAS-USZ Momentum Epithelial Cell Signaling and Secretion Research Group, University of Szeged, Dóm sqr. 10, Szeged, 6720 Hungary; 5 HCEMM-SZTE Molecular Gastroenterology Research Group, University of Szeged, Dóm sqr. 10, Szeged, 6720 Hungary; 6 Department of Internal Medicine and Hematology, Research Laboratory, Semmelweis University, Üllői út 26, Budapest, 1085 Hungary; 7 Creative Laboratory Ltd, Temesvári krt. 62, Szeged, 6726 Hungary; 8 Institute of Experimental Medicine, Eötvös Loránd Research Network, Szigony u. 43, Budapest, 1083 Hungary; 9 Wyss Institute for Biologically Inspired Engineering at Harvard University, 3 Blackfan Circle, Boston, MA 02115 USA; 10 Department of Biomedicine, Faculty of Health, Aarhus University, Høegh-Guldbergs Gade 10, 8000 Aarhus C, Denmark; 11 Institute of Applied Sciences, Department of Environmental Biology and Education, Juhász Gyula Faculty of Education, University of Szeged, Boldogasszony sgt. 6, Szeged, 6725 Hungary

**Keywords:** Acute pancreatitis, Blood–brain barrier, Ornithine, Permeability, Glycocalyx, Mitochondrial damage, Reactive oxygen stress, Cell surface charge

## Abstract

**Background:**

In severe acute pancreatitis (AP) the CNS is affected manifesting in neurological symptoms. Earlier research from our laboratory showed blood–brain barrier (BBB) permeability elevation in a taurocholate-induced AP model. Here we aimed to further explore BBB changes in AP using a different, non-invasive in vivo model induced by l-ornithine. Our goal was also to identify whether l-ornithine, a cationic amino acid, has a direct effect on brain endothelial cells in vitro contributing to the observed BBB changes.

**Methods:**

AP was induced in rats by the intraperitoneal administration of l-ornithine-HCl. Vessel permeability and the gene expression of the primary transporter of l-ornithine, cationic amino acid transporter-1 (*Cat-1*) in the brain cortex, pancreas, liver and lung were determined. Ultrastructural changes were followed by transmission electron microscopy. The direct effect of l-ornithine was tested on primary rat brain endothelial cells and a triple co-culture model of the BBB. Viability and barrier integrity, including permeability and TEER, nitrogen monoxide (NO) and reactive oxygen species (ROS) production and NF-κB translocation were measured. Fluorescent staining for claudin-5, occludin, ZO-1, β-catenin, cell adhesion molecules Icam-1 and Vcam-1 and mitochondria was performed. Cell surface charge was measured by laser Doppler velocimetry.

**Results:**

In the l-ornithine-induced AP model vessel permeability for fluorescein and *Cat-1* expression levels were elevated in the brain cortex and pancreas. On the ultrastructural level surface glycocalyx and mitochondrial damage, tight junction and basal membrane alterations, and glial edema were observed. l-ornithine decreased cell impedance and elevated the BBB model permeability in vitro. Discontinuity in the surface glycocalyx labeling and immunostaining of junctional proteins, cytoplasmic redistribution of ZO-1 and β-catenin, and elevation of Vcam-1 expression were measured. ROS production was increased and mitochondrial network was damaged without NF-κB, NO production or mitochondrial membrane potential alterations. Similar ultrastructural changes were seen in l-ornithine treated brain endothelial cells as in vivo*.* The basal negative zeta potential of brain endothelial cells became more positive after l-ornithine treatment.

**Conclusion:**

We demonstrated BBB damage in the l-ornithine-induced rat AP model suggesting a general, AP model independent effect. l-ornithine induced oxidative stress, decreased barrier integrity and altered BBB morphology in a culture BBB model. These data suggest a direct effect of the cationic l-ornithine on brain endothelium. Endothelial surface glycocalyx injury was revealed both in vivo and in vitro, as an additional novel component of the BBB-related pathological changes in AP.

**Supplementary Information:**

The online version contains supplementary material available at 10.1186/s12987-022-00308-0.

## Introduction

Acute pancreatitis (AP) is a serious gastroenterological inflammatory disease with a high mortality rate [1]. A central problem in the treatment of AP patients is that there is still no specific therapy for this disease and its complications [2, 3]. A better understanding of AP and the related comorbidities would improve the disease outcome reducing the rate of organ failure and mortality [4]. In the most severe form of AP mortality is high due to the serious damage of the exocrine pancreas, and systemic inflammation caused by the high level of secreted cytokines and other toxins in the circulation causing multiple organ failure [5, 6]. Among the complications, the central nervous system (CNS) is affected in 10% of all cases during severe AP leading to pancreatic encephalopathy [7, 8]. Disorientation, agitation, hallucinations, slow reaction time and apathy are major indicators of CNS involvement. Postmortem tissue examination reveals diffuse demyelination, edema, neuronal loss and hemorrhagic stroke in the brain, while MRI studies confirm lesions in the white matter [7]. Since the mortality rate is 50% among these patients, it is crucial to describe and identify the most important factors and changes leading to this condition [1, 8]. Recently it was found that in all forms of AP disturbance in consciousness might develop manifesting in confusion, delirium and convulsion both in alcohol-induced and non-alcohol related AP [9].

Although case studies and animal models of AP have revealed several aspects about the course of the pathomechanism, further studies are needed to fully describe this disease and its comorbidities [10]. Invasive models of pancreatitis have been developed since the 1970’s [10]. One of the surgical methods is the injection of taurocholic acid into the pancreatic duct [11]. Our research group described for the first time that in taurocholate induced AP blood–brain barrier (BBB) permeability was elevated in rats in parallel with high levels of cytokines [12]. Later new, non-invasive animal AP models were introduced. Intraperitoneal injection of cationic amino acids such as l-arginine at high concentration induces AP [13]. l-arginine is a major member of several metabolic pathways including the urea cycle, therefore l-ornithine, a metabolite of arginine, and another cationic amino acid, l-lysine, were also tested and demonstrated to generate AP in rats [14, 15]. In these models NF-κB activation, mitochondrial injury, nitric oxide (NO) and calcium-signaling were described to contribute to the development of AP [14–18]. In the l-ornithine-induced AP model, pancreatic edema is visible already after 6 h of the treatment, trypsin activity increases, pancreatic proinflammatory cytokine levels are elevated, NF-κB activation and tissue necrosis are detectable [14, 19]. Among the cationic amino acid injection methods, the most effective and most reproducible model proved to be the l-ornithine induced AP model, which by reproducing several laboratory and morphological signs is most similar to the human disease.

Brain capillary endothelial cells form the functional basis of the BBB creating a dynamic interface between the CNS and the periphery. These brain microvessel endothelial cells possess unique features, such as tight intercellular junctions, specialized nutrient and efflux transport systems, and negatively charged surface glycocalyx to protect the CNS from toxic insults, provide nutrients, and maintain the ionic homeostasis for brain functions [20]. BBB integrity, transporter activity, surface glycocalyx thickness and composition of brain endothelial cells are altered in several disease pathologies, like epilepsy, dementia, trauma, hypoxia, infections and inflammation, which alone or in combination can lead to clinical and behavioral manifestations in both patients and in animal models [21–23].

Our hypothesis is that BBB dysfunction and barrier damage play a major role in the development of CNS complications during severe AP in patients, and this can be studied by using animal models of severe AP. In this study we aimed to characterize and describe BBB integrity and morphology changes in the l-ornithine-induced AP model in rats. Expression levels of cationic transporter-1 (*Cat-1*) as primary transporter of l-ornithine in the brain and other organs was also studied to identify tissue specific changes. Our second main goal was to investigate the direct effect of l-ornithine on culture BBB models to reveal if this cationic amino acid can directly damage brain endothelial cells and contribute to the BBB alterations in the rat model additionally to the known pathogenic factors of AP.

## Materials and methods

### Materials

All reagents were purchased from Sigma-Aldrich Ltd. (belonging to Merck Group, Darmstadt, Germany) unless otherwise indicated. A table providing catalog numbers and source information of antibodies used in the present study is shared in the Additional file 1: Table S1.

### Animals

All animals were handled in accordance with approved procedures as defined by the EU Directive 2010/63/EU and all animal work was approved by the regional Station for Animal Health and Food Control Csongrád County, Hungary (project license: XVI/834/2012 for M.A.D., XVI/3554/2020 for F.R.W. and XII/955/2012 for Z.R.). Male Wistar rats were housed in conventional animal facilities of the University of Szeged and the Biological Research Centre, Szeged according to EU regulations. Animals were kept under standard conditions (22–24 °C, 12 h light–dark cycle) with regular rodent chow diet and water available ad libitum. For the isolation of primary cells, organs were harvested after the euthanasia of rats with CO_2_ inhalation.

### Induction of acute pancreatitis in rats

The non-invasive acute necrotizing pancreatitis model used in the present paper was described earlier in detail [14, 17]. Male Wistar rats weighing 200–250 g (n = 12) were injected intraperitoneally (i.p.) with 3 g/kg l-ornithine-HCl (pH = 7.4, 30%), to induce homogenous and reproducible AP pathology within 24 h. Control animals received equal volume of physiological saline solution (n = 12). To decrease dehydration, 1.5 ml Ringer-lactate solution was injected subcutaneously. To set up a humane endpoint a point scale system was developed based on our experience and literature data [24, 25]. Accordingly, animals were monitored every 2–3 h and their general state was assessed (fur, position, motility, awareness, startling reaction scored from 0 to 2). If animals reached a total sum of score above six, their level of distress was considered too high and a humane endpoint was introduced with the i.p. injection of 150 mg/kg pentobarbital.

### In vivo permeability experiments

The microvessel permeability was investigated in the brain, pancreas, liver and lung of rats in both treatment groups. One day after the induction, when acute necrotizing pancreatitis was developed, the extravasation of permeability markers, sodium fluorescein (SF, 376 Da) and Evans blue-labeled albumin (EBA, 67 kDa) was tested [26, 27]. Solutions of 2% containing both dyes were given to the animals intravenously (tail vein). After 30 min, deep anesthesia was induced by sodium pentobarbital (50 mg/kg i.p.) and animals were perfused with saline. Tissue samples from the brain cortex, pancreas, liver and lung were collected, weighed and stored frozen. To measure dye concentrations tissue samples were homogenized in 15% trichloroacetic acid and were centrifuged at 10,000 × g for 10 min at 4 °C. Concentrations of the two different dyes from the supernatants were measured using a spectrofluorometer (Photon Technology International Inc., Birmingham, NJ, USA). The fluorescence emission of SF was measured at 525 nm after the excitation at 440 nm. Absorbance of EBA was determined at 620 nm. Extravasation was calculated as ng tracer / mg tissue.

### Transmission electron microscopy

Ultrastructural changes in the acute necrotizing pancreatitis and control groups (n = 4 / group) was investigated with transmission electron microscopy (TEM). Rats were anesthetized with sodium pentobarbital (150 mg/kg), then were transcardially perfused with 0.9% NaCl in 0.1 M phosphate buffer. Animals were then perfused with 0.1 M phosphate buffer containing 4% paraformaldehyde (PFA), 2.5% glutaraldehyde and 2% Alcian blue. Brains and pancreata were removed and post-fixed with 4% PFA in 0.1 M phosphate buffer overnight at 4 °C. Three mm thick tissue blocks of a half brain containing the frontal cortex region or 2 × 5 × 7 mm tissue blocks of the pancreas were cut out by blade and washed in 0.1 M phosphate buffer. Coronal brain Sects. (50 µm) or pancreas Sects. (60 µm) were obtained by a vibrating microtome (VT1000S; Leica Microsystems, Wetzlar, Germany). Sections were postfixed with 1% OsO_4_ for 1 h, rinsed with distilled water, dehydrated in graded ethanol, en bloc stained with 2% uranyl acetate in 70% ethanol for 30 min, finally embedded in Taab 812 (Taab Equipment Ltd., Aldermaston, UK). Following overnight polymerization at 60 °C, ultrathin sections were obtained using a Leica UCT ultramicrotome (Leica Microsystems). The digital pictures were taken on a Hitachi 7100 electron microscope (Hitachi, Tokyo, Japan). Brightness and contrast were adjusted if necessary using Adobe Photoshop CS3 (Adobe Inc., San Jose, CA, USA). A total of 28–28 images of the sham (control) and AP groups were analyzed and morphological changes in endothelial cells of brain cortical capillaries are summarized in Table 1.

**Table 1:** Summary of changes in the ultrastructure of capillary endothelial cells in the brain cortex of rats with acute pancreatitis induced by l-ornithine

Brain capillary endothelial cells		Control	Acute pancreatitis
*Number of images analyzed*		28	28
Luminal membrane	Smooth	73%	62%
	Protrusions	27%	38%
Glycocalyx	Intact	96%	31%
	Discontinuous	4%	69%
Tight junctions	Intact	100%	86%
	Discontinuous	0%	14%
Mitochondria	Intact	100%	26%
	Altered	0%	74%
Vacuolization	Present	32%	85%
Basal membrane	Intact	94%	44%
	Altered or dye leakage	6%	66%

Values are shown in percentage of the analyzed images in the groups

### Assessment of the gene expression of cationic amino acid transporter-1 (*Cat-1*)

The mRNA expression of *Cat-1* was measured using quantitative real-time PCR (qPCR) following RNA isolation and cDNA synthesis from brain microvessels, brain cortex, pancreas, liver and lung samples (n = 3–6). Tissue samples were collected at 3 different time points after l-ornithine injection. Rats were anesthetized with pentobarbital (50 mg/kg), cervically dislocated and decapitated. The abdomen of the animals was quickly opened and ~ 100 mg tissue pieces from the pancreatic head were dissected and snap frozen in liquid nitrogen within 90 s to avoid damage of pancreatic tissue. Next, samples from the liver and the lung were collected. After opening the skull, the whole brain was collected, the cerebellum was removed and brain cortex samples were dissected. Following the removal of white matter pieces, the rest of the brain tissue was used for brain microvessel isolation based on our recently published protocol [28]. All samples were snap frozen and homogenized in TRI Reagent (Molecular Research Center Inc., Cincinnati, OH, USA) with an electric homogenizer (T8.01, IKA Labortechnik, Staufen im Breisgau, Germany). Isolated brain microvessels were homogenized with a glass homogenizer.

The homogenates were used for isolation of total RNA. Briefly, after phase separation with chloroform, RNA was precipitated with isopropanol and cleaned with a subsequent precipitation with lithium chloride (LiCl). The precipitated RNA was dissolved in 200 µl sterile distilled water, reprecipitated with additional 60 µl of 8 M LiCl and incubated on ice for 30 min. The precipitated RNA was collected with centrifugation and washed twice with ice-cold 80% ethanol. After air-drying RNA samples were dissolved in 40 µl of sterile distilled water and were measured using NanoDrop 1000 spectrophotometer (Thermo Fisher Scientific Inc., Waltham, MA, USA). RNA quality was also confirmed by agarose gel electrophoresis.

After DNase treatment (Thermo Fisher Scientific Inc.), 1 μg RNA from each sample was transcribed to complementary DNA with Maxima First Strand cDNA Synthesis Kit (Thermo Fisher Scientific Inc.) following the manufacturer’s instructions. To perform qPCR, gene-specific and exon/exon junctions covering oligonucleotide primer pairs were used from The Universal Probe Library Assay Design Center (Merck). The following primer pairs were used with FAM-TAMRA labelled universal probes: for Cat-1/Slc7a1 transporter (gene ID: NM_013111.3) fw 5’-GGAGCTCTGGGCCTTCAT-3’; rv 5’-CTTGCCACGCTGGATGTA-3’; probe #20; for hypoxanthine phosphoribosyltransferase 1 (Hprt-1; gene ID: NM_012583.2) fw 5’-GACCGGTTCTGTCATGTCG-3’; rv 5’-ACCTGGTTCATCATCACTAATCAC-3’; probe #95. qPCR was performed with iQ Supermix (Bio-Rad Laboratories Inc., Hercules, CA, USA) in a CFX96 Real-Time PCR Detection System (Bio-Rad Laboratories Inc.). After heat activation at 95 °C for 3 min the following cycling conditions were applied: denaturation for 30 s at 93 °C, annealing and polymerization for 60 s at 60 °C and registration of fluorescent signals (50 cycles). Data was processed with the CFX Manager software (Bio-Rad Laboratories Inc.). Relative gene expression levels were normalized to the endogenous control gene *Hprt-1* (ΔCt). Then ΔΔCt was calculated in comparison to the lowest relative expression of *Cat-1/Slc7a1* in the liver samples from the vehicle-treated group. Fold changes were calculated using the 2^−ΔΔCt^ formula.

### Primary rat brain cell cultures

Primary rat brain endothelial cells and primary brain microvessel pericytes along with primary brain astroglia cells were isolated according to our well established and characterized method [27, 29, 30]. Reagents for the isolation are listed in our previous paper [31]. Primary cell based culture models are widely used and suitable systems to investigate the pathology, pharmacology and physiology of the BBB in vitro [32]. For permeability and barrier integrity studies primary rat brain endothelial cells were co-cultured with primary rat brain pericytes and glial cells on cell culture inserts to induce BBB properties. All surfaces for primary brain endothelial cells were coated with collagen type IV and fibronectin (100 µg/ml each in sterile distilled water). To culture pericytes and astrocytes, collagen type IV (100 µg/ml in sterile distilled water) coating was used. Cell culture medium during all experiments’ growth period contained 10% plasma derived serum (PDS, First Link UK Ltd., Birmingham, UK), 5 μg/ml insulin, 5 μg/ml transferrin, 5 ng/ml sodium selenite (ITS, Thermo Fisher Scientific Inc.), 1 ng/ml basic fibroblast growth factor, 10 mM HEPES, 100 µg/ml heparin and 50 µg/ml gentamycin in DMEM/F12 (Thermo Fisher Scientific Inc.). In all experiments 24 h before treatment cells received 550 nM hydrocortisone to tighten barrier properties [31, 32].

### Cell viability assays

#### MTT assay

For this method primary rat brain endothelial cells were seeded on 96-well plates (Corning Costar, Corning, NY, USA) at a cell number of 5 × 10^4^ cells/well. Cells reached confluency within 3–4 days, then cultures were treated with 1–40 mM l-ornithine or d-ornithine. Triton-X100 (1%) was used to induce cell death. Conversion of the yellow MTT dye (3-(4,5-dimethylthiazol2-yl)-2,5-diphenyltetrazolium bromide) to blue formazan crystals reflects the metabolic activity: wells with living cells turn blue and wells with less viable cells stay more yellow. After 24 h treatment, solutions were removed and cells were incubated with 0.5 mg/ml MTT dye in phenol red-free DMEM/F12 (Thermo Fisher Scientific Inc.). Cell cultures were observed during this period every 30 min until maximum crystal conversion occurred (~ 1.5–2 h). Formazan crystals were then dissolved in dimethyl sulfoxide, and absorbance was measured with a multiwell plate reader (Fluostar Optima, BMG Labtechnologies, Offenburg, Germany) at 592 nm. Cell viability was calculated as a percentage of the medium-treated control group, where the maximum dye conversion was observed.

### Impedance measurement

Using a label-free, noninvasive, impedance based technique (RTCA SP, Agilent, Santa Clara, CA, USA) confluency, morphology and barrier integrity of primary rat brain endothelial cells can be monitored in real time. This technique correlates linearly with the cell number, adherence, growth and viability of the cells [27, 33]. This method is also a good verification for end-point colorimetric tests, such as MTT assay [33]. Here primary rat brain endothelial cells at a cell number of 5 × 10^4^ cells/well were seeded on special 96-well plates with embedded gold electrodes (E-plate, Agilent). First, background impedance was measured with the addition of 50 µl cell culture medium, then cells were added and growth was followed. Once cells reached the plateau phase of growth they were treated with 1–40 mM l-ornithine or d-ornithine, or with the reference compound 1% Triton-X100 detergent to induce cell death. Effects of the treatment was followed for 24 h.

### Barrier integrity tests

For these assays the triple co-culture model of the BBB was established from primary rat brain endothelial cells, primary rat brain pericytes and glial cells on cell culture inserts (Transwell inserts: 12 mm diameter, 1.12 cm^2^ surface, 0.4 µm membrane pore size, polyester membrane; Corning Costar).

### Transendothelial electrical resistance measurement

To follow the development of barrier properties of primary rat brain endothelial cells in co-culture transendothelial electrical resistance (TEER) was measured every second day before the medium change on a heating pad set to 37 °C using an EVOM Volt/Ohm Meter with an STX-2 electrode (World Precision Instruments, Sarasota, FL, USA). The TEER was always calculated relative to the surface of the cell culture insert (Ω × cm^2^) and the background of cell-free inserts was subtracted from the measured values (120 Ω × cm^2^). TEER was measured 24 h after 20 mM l-ornithine or d-ornithine treatment, culture medium-treated cells served as control.

### Permeability studies

Directly after the 24 h 20 mM l-ornithine or d-ornithine treatment and the resistance measurements, the BBB model was tested for permeability of two fluorescent dyes, SF (10 µg/ml) and EBA (170 µg/ml EB and 10 mg/ml bovine serum albumin, BSA) in Ringer-Hepes buffer exactly as described previously [34]. Permeability of cell-free inserts was also tested and used later to calculate the permeability coefficient for endothelial monolayers (P_e_) [34] for each treatment condition.

### Immunohistochemistry

To investigate the direct effect of l-ornithine treatment on junctional integrity primary brain endothelial cells were stained for four membrane and membrane associated junctional proteins: tight junction (TJ) molecules claudin-5 and occludin, and junctional associated cytoplasmic linker proteins zonula occludens-1 (ZO-1) and β-catenin. Directly after the permeability measurements, inserts containing cells were rinsed once in Ringer-Hepes buffer and were fixed with 1% PFA-phosphate buffered saline (PBS) solution for 15 min at room temperature. Cells were stored in 0.1% sodium azide-PBS until staining. Cells were also cultured on collagen type IV coated glass cover slips, treated when reaching confluency, then fixed and stored similarly. First, cells were permeabilized with 0.1% Triton-X100 in PBS for 15 min at 4 °C. Cells were then washed in PBS and were treated with 1% BSA-PBS for 1 h at room temperature to block the unspecific binding sites. Incubation with rabbit primary antibodies for claudin-5 (SAB4502981, 1.6 µg/ml), occludin (71–1500, 1.25 µg/ml, Thermo Fisher Scientific Inc.), ZO-1 (61–7300, 0.625 µg/ml, Thermo Fisher Scientific Inc.) and β-catenin (C2206, 1.6 µg/ml) lasted overnight at 4 °C in blocking buffer. The next day, cells were washed in PBS and were incubated with the secondary antibody sheep anti-rabbit-Cy3 (C2306, 2.5 µg/ml) for 1 h at room temperature in PBS. After this step, cells were washed again with PBS, then once with distilled water and samples were mounted with Fluoromount G mounting medium (Southern Biotech, Birmingham, AL, USA). Pictures were taken with a TCS SP5 confocal laser scanning microscope (Leica Microsystems, Wetzlar, Germany). Image merging was performed using the ImageJ software (NIH, Bethesda, MD, USA). The fluorescently labeled images were also analyzed using the Matlab software (R2019a, MathWorks, Inc., Natick, MA, USA). The intracellular areas (cytoplasm) of the cells on each image were defined by a freehand region of interest (ROI) determination, performed by a blinded experimenter. These ROIs were subtracted from the original image, thus the resulting image contained only the immunostaining at the intercellular junctions. Using the threshold of the original image, both the image of the cytoplasm and the TJs were converted to binary images (BI). The BIs were used as masks to determine the cumulative intensities of the original pixels for both cytoplasmic and TJ images. The ratio of the cell membrane and cytoplasm intensities for each image was calculated and used for the statistical analysis.

Staining for the intercellular adhesion molecule-1 (mouse anti-Icam-1, MA5407, 2 µg/ml, Thermo Fisher Scientific Inc.) and the vascular cell adhesion molecule-1 (mouse anti-Vcam-1, 13–1060-82, 2 µg/ml, Thermo Fisher Scientific Inc.) were performed similarly as written above. Experiments were finished with incubation in goat anti-mouse-Alexa488 secondary antibody (A-11029, 4 µg/ml, Thermo Fisher Scientific Inc.). Analysis was performed using the Olympus FV1000 confocal microscope (Olympus Corporation, Tokyo, Japan). Image merging and analysis measuring total mean gray value was done with the ImageJ software (NIH).

### Measurement of reactive oxygen species and nitric oxide production

Both methods to measure reactive oxygen species (ROS) is based on cell-permeable, inherently non-fluorescent dyes, which are converted into a fluorescent form when they enter the cells and detect free oxygen species. All kinds of ROS are detected by the 2',7'-dichlorodihydrofluorescein diacetate (H_2_DCFDA, Thermo Fisher Scientific Inc.), while NO production is measured by the 4-amino-5-methylamino-2',7'-difluorofluorescein diacetate (DAF-FM diacetate, Thermo Fisher Scientific Inc.). Primary rat brain endothelial cells were seeded at a cell number of 5 × 10^4^ onto 96-well plates with black wall and transparent bottom (Corning Costar, USA). After reaching confluency, cells were treated with 20 mM l-ornithine for 24 h. The assays were performed as previously described in our publications [30, 35]. Reference compounds hydrogen peroxide (H_2_O_2_, 100 µM) for ROS and sodium nitroprusside (SNP, 100 µM) for NO production were used to validate the assays.

### Mitochondrial network staining

Cellular stress influences the basic functions of mitochondria and the mitochondrial network within a cell. Therefore, we aimed to visualize this network using the Mitotracker Green dye (Thermo Fisher Scientific Inc.) according to the manufacturer’s instructions. Since this dye cannot be fixed, we performed live cell imaging. Primary brain endothelial cells were grown on glass bottom 35 mm Petri dishes (MatTek Corporation, Ashland, MA, USA) until confluency. Cells were treated with 20 mM l-ornithine for 24 h, or a mitochondrial network decoupling agent, carbonyl cyanide 3-chlorophenylhydrazone (CCCP, 5 µM) for 30 min. Control cells received cell culture medium. After treatment cells were stained in Ringer-Hepes buffer with Mitotracker Green (0.1 µM) and Hoechst 33,342 nucleus dye (0.6 µg/ml) for 30 min in the cell culture incubator. Cells were then washed three times and fluorescent microscopy pictures were taken on living cells in the Ringer Hepes buffer using the Olympus FV1000 confocal microscope (Olympus Corporation). Image analysis was performed using the ImageJ software (NIH). Intracellular mitochondrial network integrity was evaluated using the Matlab software (R2019a, MathWorks, Inc.). The fluorescent image of the mitochondrial network was shrinked to a skeletal web. Then, the number of the branchpoints were determined and the average branch length was calculated. Lower average object length represents a disorganized and disassembled mitochondrial network.

### Transmission electron microscopy of the BBB co-culture and image analysis

In addition to the in vivo TEM studies, we also aimed to visualize the direct effect of l-ornithine treatment on the ultrastructure of primary rat brain endothelial cells. The triple co-culture BBB model was established and treated with 20 mM l-ornithine for 24 h, as described earlier. After treatment, cells on both surfaces of the membrane of the inserts were washed quickly in warm PBS, then rinsed with the fixative containing 3% PFA and 0.5% glutaraldehyde (GA) in cacodylate buffer (0.05 M sodium cacodylate, 0.25 M saccharose in filtered distilled water, pH of 7.4) at room temperature. It was followed by fixation with a new portion of the fixative supplemented with 1% Alcian blue for 30 min at 4 °C. This cationic dye is capable of staining the highly negatively charged surface glycocalyx in cell cultures as well, similarly to the in vivo studies. Then the inserts were washed with cacodylate buffer and were processed for TEM analysis as written for the tissue samples. First, the membranes with the cells on their two sides were removed from their support and placed into 24-well chamber slides, postfixed in 1% OsO_4_ for 30 min at room temperature, then rinsed with distilled water. Dehydration and embedding in Taab 812 (Taab Equipment Ltd.) were performed as written in the in vivo TEM section. Since the whole membrane was embedded the small resin blocks suitable for sectioning were cut with a saw. Ultrathin sections were cut perpendicularly for the membrane using a Leica UCT ultramicrotome (Leica Microsystems) and examined using a Hitachi 7100 transmission electron microscope (Hitachi Ltd.). Electron micrographs were made by Megaview II (lower resolution, Soft Imaging System, Münster, Germany). Brightness and contrast were adjusted if necessary using Adobe PhotoshopCS3 (Adobe Inc.). A total of 29 images from the control and 48 of the l-ornithine treated groups were analyzed, and ultrastructural changes in cultured brain endothelial cells are shown in Table 2.

**Table 2:** Summary of changes in the ultrastructure of l-ornithine treated cultured brain capillary endothelial cells

Cultured brain endothelial cells		Control	l-ornithine
*Number of images analyzed*		29	48
Luminal membrane	Smooth	74%	67%
	Protrusions	26%	33%
Glycocalyx	Continuous	89%	29%
	Discontinuous	11%	71%
Tight junctions	Intact	100%	78%
	Discontinuous	0%	22%
Mitochondria	Intact	97%	53%
	Altered	3%	47%
Vesicular profiles / image	Luminal membrane	5.9 ± 4.3	8.7 ± 4.5^*^
	Abluminal membrane	2.2 ± 1.5	5.9 ± 3.8^***^
Vesicular profiles ratio	Luminal membrane	73%	60%
	Abluminal membrane	27%	40%
Vacuolization	Present	14%	27%

Values are shown in percentage or ratio of the analyzed images in the groups. Vesicular profile: mean ± SD, unpaired t-test; *, p < 0.05; ***, p < 0.001

### Zeta potential measurements

To assess the direct, acute effect of l-ornithine treatment on the surface charge (zeta potential) of primary rat brain endothelial cells, we used the dynamic light scattering method measured by a Zetasizer NanoZS instrument (Malvern Panalytical, UK). The measurement was performed similarly as we published before [33, 36]. Briefly, confluent layers of cells in a 35 mm or 60 mm Petri dish were quickly and gently trypsinized and counted. For each condition aliquots of 10^5^ cells/microcentifuge tube were prepared. Cells in microtubes received 5–20 mM concentrations of l-ornithine in PBS, then zeta potential was immediately measured. Cell suspensions were directly injected into the folded capillary zeta cell and surface charge measurement was performed. Values presented in mV were calculated by the software of the instrument.

### Statistical analysis

Data are presented as means ± SEM. Statistical significance between treatment groups was determined using the Graph Pad Prism 5.0 Software (GraphPad, USA) using unpaired t- test, one-way and two-way Anova analyses with Bonferroni post-tests. All experiments were repeated at least twice and the number of total biological and technical parallels were always at least three. Changes were evaluated as statistically significant when p < 0.05.

## Results

### Pancreas and brain vessel permeability is elevated in l-ornithine-induced acute pancreatitis in rats

**Fig. 1:**
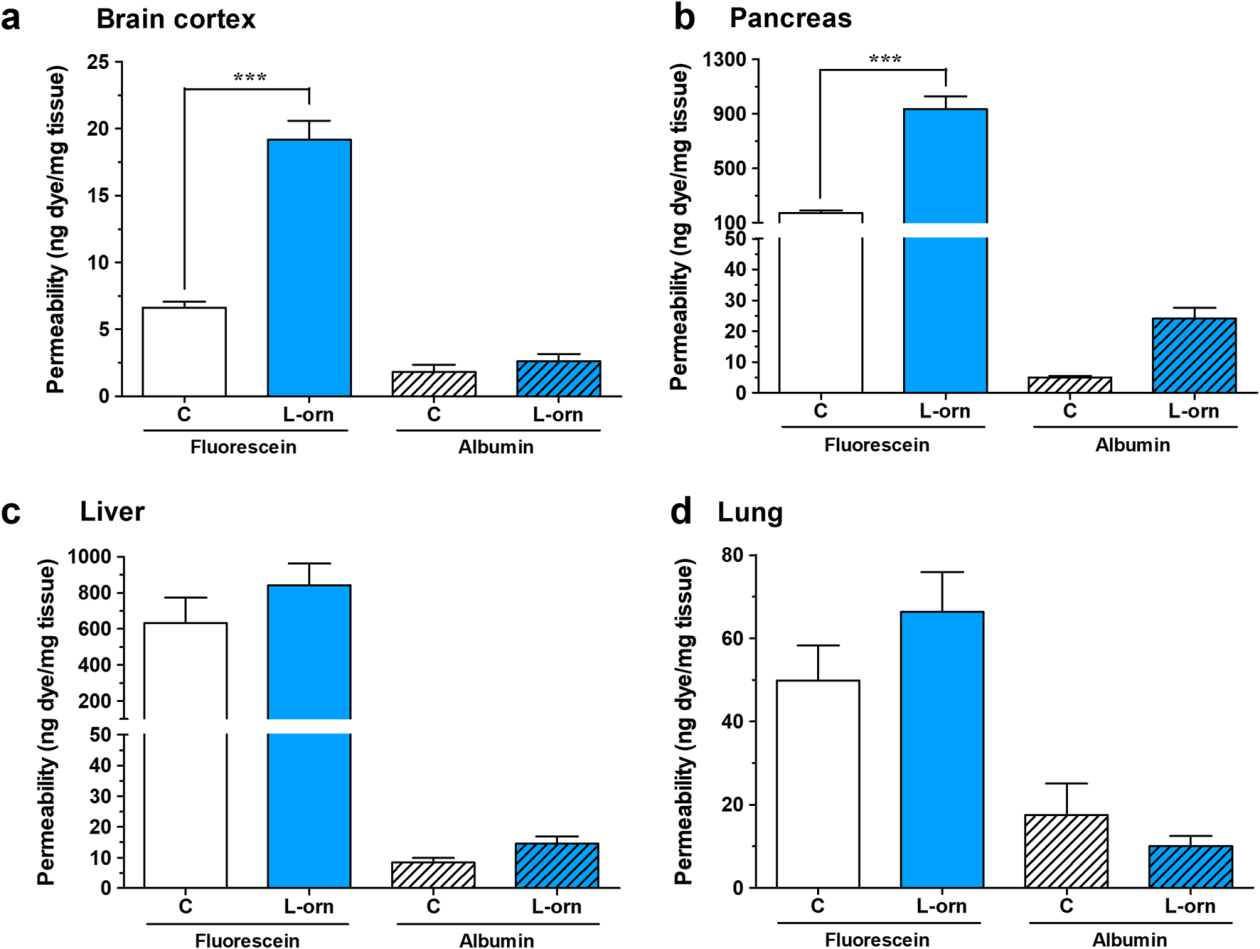
Extravasation of sodium fluorescein and Evans blue-labeled albumin in the **a** brain cortex, **b** pancreas, **c** liver and **d** lung of rats injected with saline and animals with l-ornithine-induced acute pancreatitis. Values are given as nanogram dye permeability / miligram tissue and are presented as means ± SEM, n = 6. Statistical analysis: two-way Anova followed by Bonferroni post-test, ***, p < 0.001

The permeability of vessels was analyzed in the brain, pancreas, liver and lung (Fig. 1). In the AP group, the extravasation of sodium fluorescein was significantly elevated in the brain cortex and in the pancreas, while no change was seen for the albumin permeability. Vessel permeability in the liver and lung was not changed for the marker molecules. The lowest basal permeability was observed in the brain, followed by the lung and pancreas, while the highest vascular leakage was seen in the liver (Fig. 1).

### Induction of acute pancreatitis by l-ornithine increases the expression of the cationic amino acid transporter-1 (Cat-1) in the brain and the pancreas of rats

**Fig. 2:**
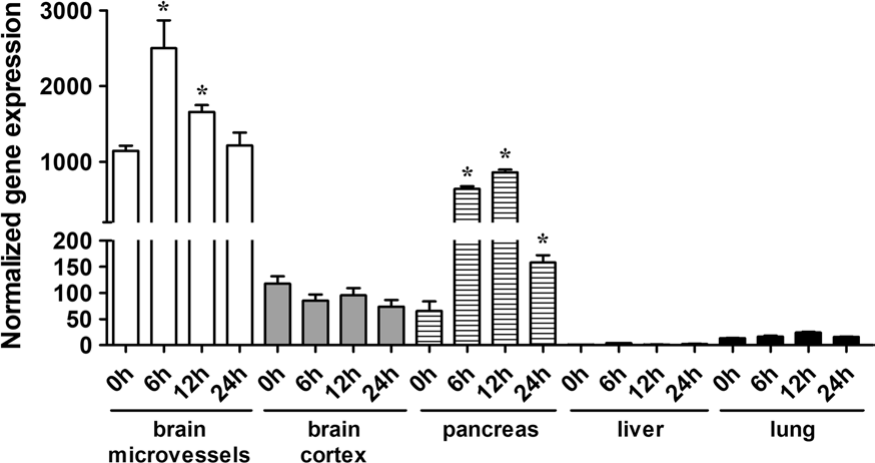
Relative gene expression changes of cationic amino acid transporter-1 (*Cat-1/Slc7a1*) measured by qRT-PCR in different organs from rats with acute pancreatitis. Expression levels were normalized to *Hrpt-1* mRNA expression in the liver of the untreated group. Values presented are means ± SEM, n = 3–6, one-way ANOVA, Bonferroni test, *p < 0.05 compared to the 0 h time point

Since l-ornithine is a cationic amino acid, we investigated the gene expression of the most abundant cationic amino acid transporter, *Cat-1/Slc7a1*, in different organs (Fig. 2). The relative basal mRNA level of *Cat-1* was the highest in brain microvessels. Gene expression of *Cat-1* was elevated in the brain microvessel and pancreas samples of rats with AP. In brain microvessels, significantly higher mRNA level was found 6 and 12 h after l-ornithine injection compared to the basal level (0 h). In the pancreas, elevated *Cat-1* gene expression was detected in the AP group at all the three time points. *Cat-1* levels were low in brain cortical tissue and lung samples, and no significant change in the gene expression was seen during the development of AP. The lowest *Cat-1* expression was detected in the liver (close to detection limit).

### Glycocalyx damage, edema and junctional disturbances occur at the ultrastructural level in the brain and pancreas microvessels during acute pancreatitis in rats

Tissue samples from the pancreas and the brain of AP rats were also analyzed using TEM. To visualize the negatively charged glycocalyx, the animals were perfused with a solution containing Alcian blue, a cationic dye.

**Fig. 3:**
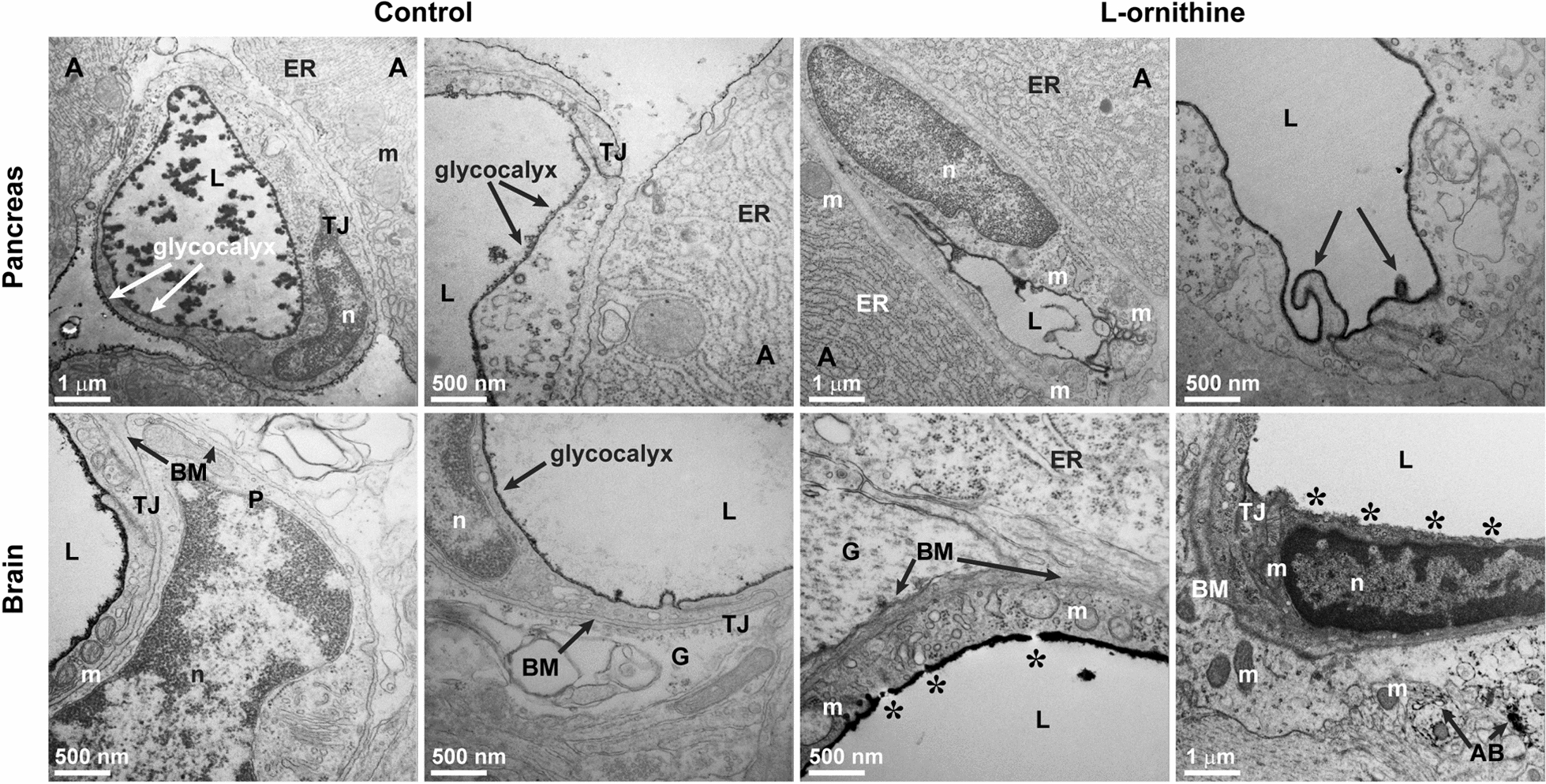
Representative transmission electron micrographs of pancreas and brain from rats injected with saline (control) and animals with acute pancreatitis induced by l-ornithine (n = 4). The negatively charged surface glycocalyx was labeled with the cationic dye Alcian blue. *A* acinar cells, *AB* Alcian blue, *BM* basal membrane, *ER* endoplasmic reticulum, *G* glial endfeet, *L* vessel lumen, *m* mitochondria, *n* nucleus, *P* pericyte, *TJ* intercellular tight junction. Black arrows in the l-ornithine-treated pancreas group point to plasma membrane deformities on the luminal surface of endothelial cells. Stars in the l-ornithine-treated brain group label discontinuities of the luminal surface glycocalyx. Scale bars: 500 nm or 1 µm

In the vehicle-treated animals, the glycocalyx lining the lumen of microvessels was clearly visible and formed a continuous coverage in the pancreas (Fig. 3). Intercellular junctions were continuous and intact, and there were no ultrastructural changes in any major cellular components, such as mitochondria, nuclei or basal membrane. The morphology of the perivascular acinar cells in the pancreas showed no alterations. In the AP group, we observed microvessel and endothelial cell deformities (Fig. 3) as well as severe endothelial ultrastructural damage (Additional file 1: Fig. S1). The pancreatic tissue showed signs of edema and necrosis with damaged mitochondrial structure (Additional file 1: Fig. S1).

In the brain, we noticed changes indicating barrier integrity damage in endothelial cells in AP: (i) discontinuity in the surface glycocalyx staining, (ii) appearance of the cationic Alcian blue on the abluminal side of the capillaries suggesting leaky tight junctions, (iii) damaged basal lamina, (iv) swollen glial endfeet (Fig. 3). In the endothelial cells of brain cortex capillaries, the luminal surface was mostly smooth and protrusions were present in the third of images from both healthy and AP groups (Table 1). The surface glycocalyx of brain capillaries, visualized by the cationic dye Alcian blue showed a discontinuous pattern in 69% of the cases of AP animals, while glycocalyx was intact in healthy rats. Only intact TJ profiles were found in brain capillaries from control rats. In AP most of the TJs were also intact, we found only 14% altered (discontinuous or open) TJs (Table 1). Mitochondria with altered morphology, including less-well defined or not visible cristae and swollen areas were only observed in rats with L-ornithine-induced AP. In AP vacuolization was found in 85% of endothelial cells (Table 1), a 2.5-fold increase as compared to the control group. The basal membrane showed an intact morphology in the control group, while in AP rats basal membrane thickening, uneven or irregular profile was observed along with Alcian blue leakage to the surrounding tissue in 66% of the analyzed images (Table 1), indicating BBB disruption. Other ultrastructural changes in brain capillaries in the AP group included condensation of heterochromatin in the nucleus of endothelial cells and increase in the gap between the glial endfeet and brain endothelial cells reflecting also basal lamina damage.

We found microvessel damage in both tissues on the ultrastructural level in AP, interestingly not only in the pancreas, but also in the brain. We suggest that capillary endothelial cell damage in AP can be linked to the observed permeability elevation.

### l-ornithine decreases metabolic activity and impedance of cultured brain endothelial cells

**Fig. 4:**
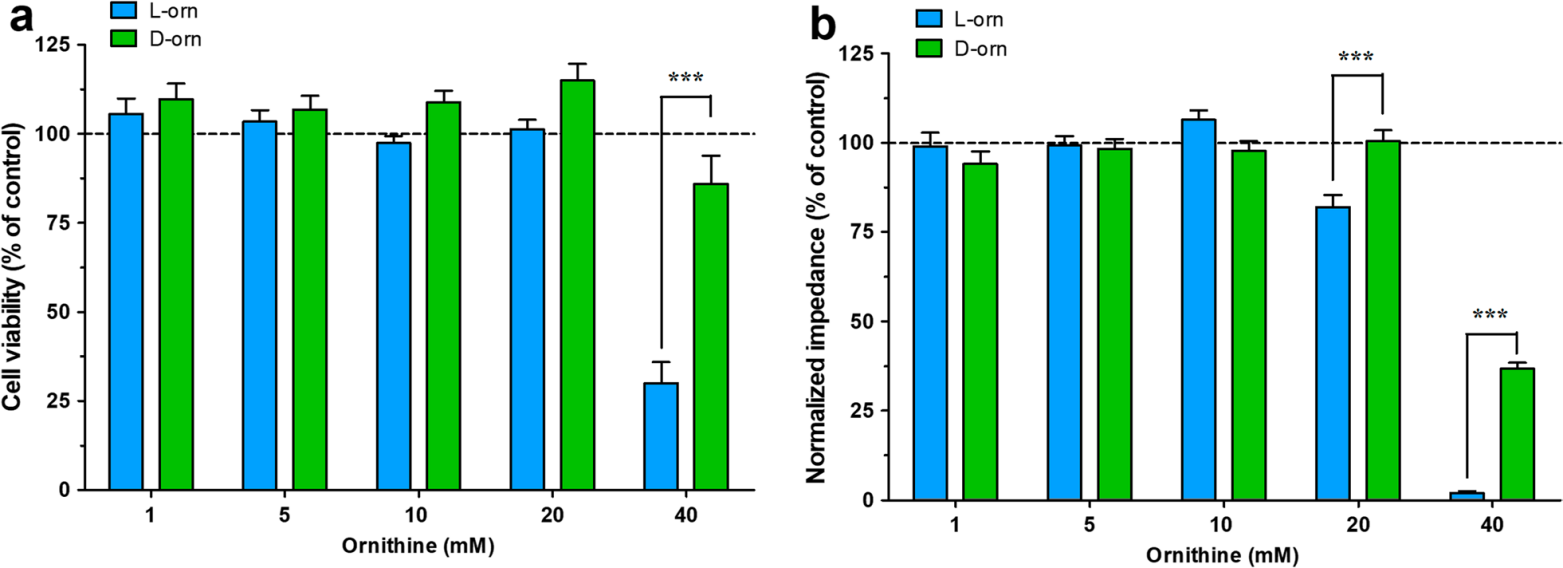
The effects of l- and d-ornithine (l-orn and d-orn, 1–40 mM, 24 h) on the cell viability of primary brain endothelial cells. Control groups received only culture medium. The bar charts show the results of **a** MTT assay, n = 4–28 and **b** real-time impedance measurement, n = 8. Values presented are means ± SEM, statistical analysis: two-way Anova followed by Bonferroni post-test, ***, p < 0.001

To explore, if l- ornithine has a direct effect on endothelial cells that might contribute to the observed increased brain extravasation and cerebral endothelial damage observed in the rat AP model, we investigated different concentrations (1–40 mM) of l- ornithine on cell viability, function and morphology of rat primary brain endothelial cells. First, the metabolic activity of primary rat brain endothelial cells was evaluated. The biologically inactive enantiomer of the amino acid, d-ornithine was also tested. MTT dye conversion decreased only at the highest, 40 mM concentration of l-ornithine after 24 h treatment, while d-ornithine had no effect on the metabolic activity of endothelial cells (Fig. 4a). The impedance measurement showed that 24 h treatment with 20 mM l-ornithine had a mild effect, and both l- and d-ornithine decreased the impedance of cell layers at 40 mM concentration. A significant difference was found between the effects of d- and l-ornithine in this assay both at 20 mM and 40 mM concentrations. Since the 40 mM concentration decreased metabolic activity and impedance, we decided to carry on all further experiments with the 20 mM concentration.

### l-ornithine decreases barrier integrity in a triple co-culture blood–brain barrier model

**Fig. 5:**
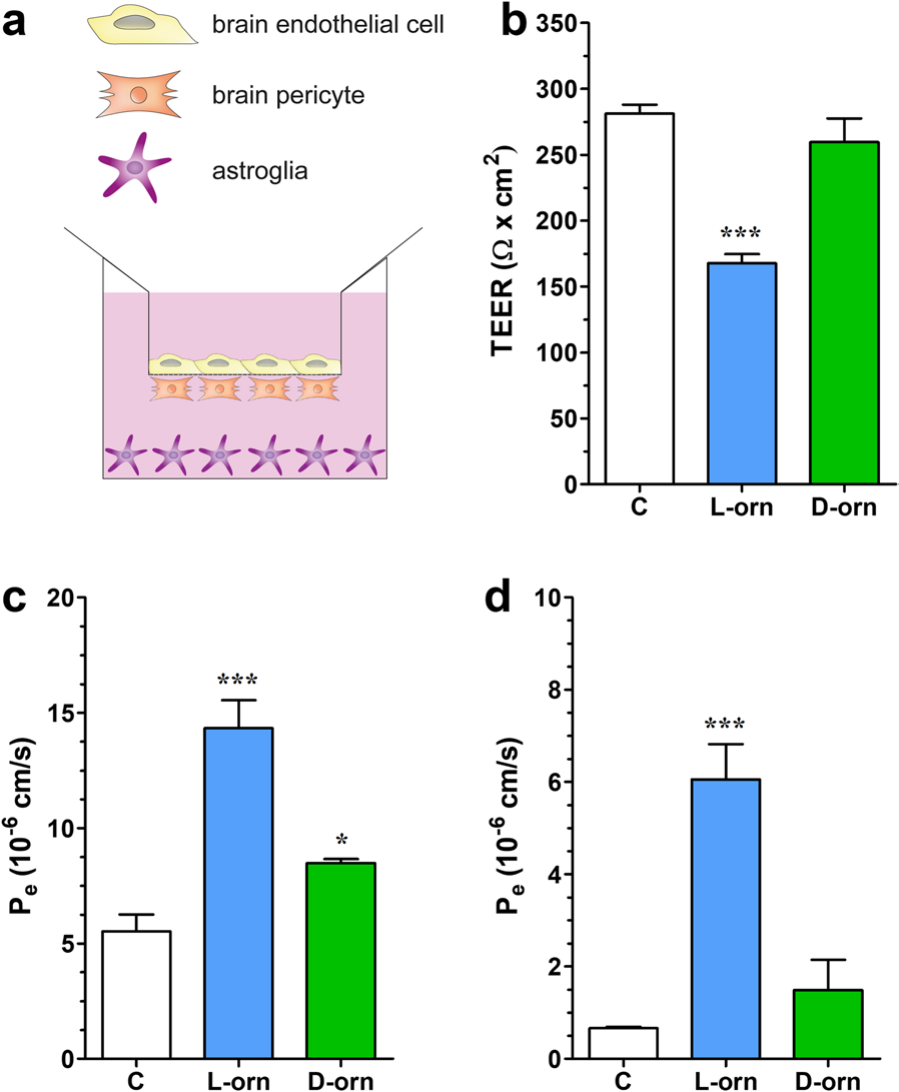
The effects of l- and d-ornithine (l-orn and d-orn, 20 mM, 24 h) on the permeability of the triple BBB co-culture model. **a** Schematic drawing of the triple BBB co-culture model assembled from primary rat endothelial cells, brain pericytes and astroglia cells. **b** Transendothelial electrical resistance (TEER) measurement across the model, n = 3. **c** Permeability for sodium fluorescein (376 Da), n = 4–5; **d** Permeability for Evans blue-labelled albumin (67 kDa), n = 4–5. Control groups (C) received culture medium. Values presented are means ± SEM. Statistical analysis: one-way Anova followed by Bonferroni post-test, *, p < 0.05; ***, p < 0.001. Permeability is expressed as endothelial permeability coefficient (P_e_)

We investigated the effects of l- and d-ornithine (20 mM, 24 h) on the barrier integrity of a primary cell-based triple co-culture BBB model. We found that l-ornithine treatment caused a decrease in the TEER along with elevated SF and EBA flux across the model compared to the control group (Fig. 5). d-ornithine treatment only showed a mild elevation in the SF permeability which was not corroborated by an elevation in EBA permeability or decreased TEER (Fig. 5). Since the effect of d-ornithine treatment was significantly milder compared to that of l-ornithine and similar to the control group both in the viability assays and in the barrier integrity studies, we decided to focus on only l-ornithine in further experiments.

### l-ornithine alters the distribution of junctional associated molecules and elevates Vcam-1 expression

**Fig. 6:**
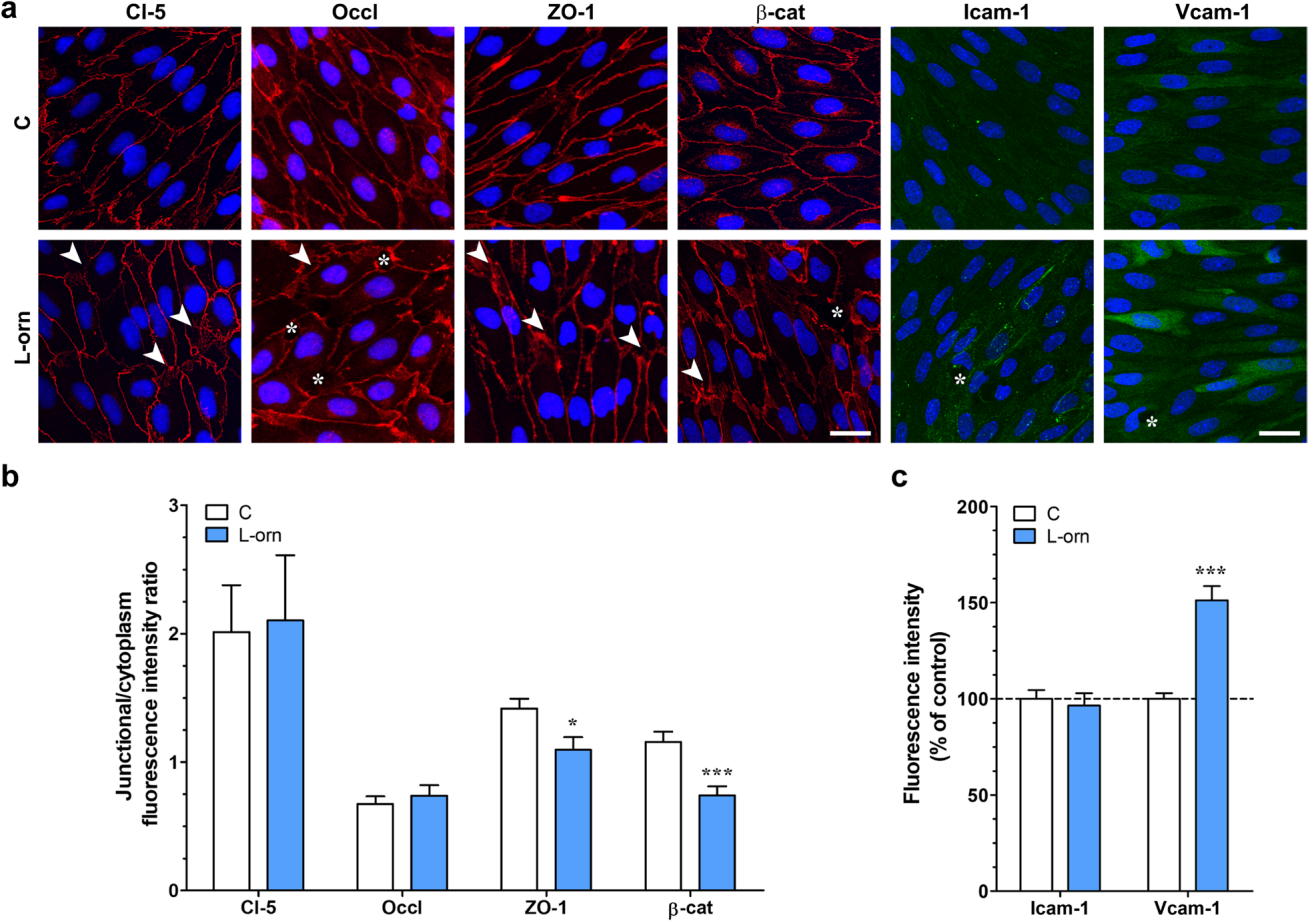
Effects of l-ornithine (l-orn, 20 mM, 24 h) on junctional morphology and adhesion molecule expression. **a** Representative immunofluorescent pictures of transmemebrane tight junction proteins claudin-5 (Cl-5) and occludin (Occl), junctional associated molecules zonula occludens-1 (ZO-1) and β-catenin (β-cat), and cell adhesion molecules intercellular adhesion molecule-1 (Icam-1) and vascular cell adhesion molecule-1 (Vcam-1). Arrows point to membrane perturbations, stars indicate monolayer discontinuity. C: control. Scale bar: 20 µm for junctional stainings and 25 µm for Icam-1/Vcam-1. **b** Ratio of the junctional and the cytoplasmic immunostaining intensities. Random pictures (4–5 images/sample), from 2–3 different experiments (n = 15–25) were compared. **c** Fluorescence intensity of cell adhesion molecules Icam-1 and Vcam-1. Random pictures (4–5 images/sample), from 2 different experiments (n = 18–28) were evaluated. Values presented are means ± SEM, statistical analysis: unpaired t-test. *, p < 0.05; ***, p < 0.001

To confirm the barrier integrity decreasing effects of l-ornithine we performed immunostaining for transmembrane (claudin-5 and occludin) and cytoplasmic linker (ZO-1 and β-catenin) junctional molecules in primary brain endothelial cells (Fig. 6a). In the control group staining of junctional proteins were located to the cell borders and the cells displayed typical, elongated morphology. After l-ornithine treatment we found discontinuity and alterations in the immunostaining of junctional proteins (Fig. 6a). Subcellular localization of the junctional molecules was analyzed with fluorescence intensity measurements (Fig. 6b). TJ associated molecules ZO-1 and β-catenin showed cytoplasmic redistribution, indicated by the decreased junctional/cytoplasmic ratio of the staining compared to the control. In addition to the changes in the junctional morphology, elevation of the Vcam-1 staining intensity was seen in the l-ornithine treated group, while no change was measured for the Icam-1 expression.

### l-ornithine treatment increased reactive oxygen species production and induced mitochondrial network disorganization in cultured brain endothelial cells

**Fig. 7:**
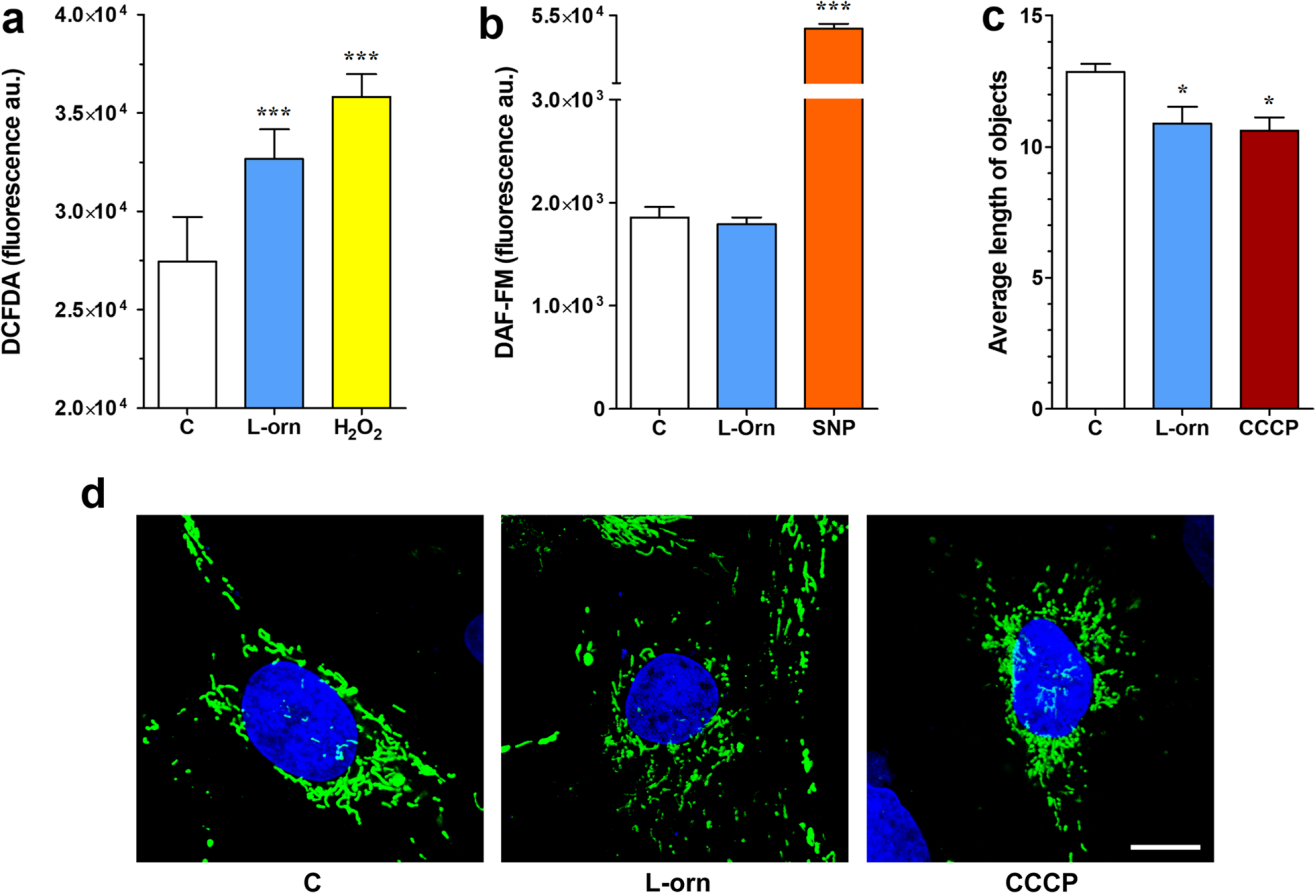
Effects of l-ornithine (l-orn, 20 mM, 24 h) on the general oxidative state of cultured primary brain endothelial cells. **a** Reactive oxygen species production measurement using the DCFDA reagent. In this assay hydrogen peroxide (H_2_O_2_, 100 µM) was used to induce oxidative stress. n = 5–12. **b** Nitric oxide (NO) production was detected by using the DAF-FM diacetate reagent. In this assay sodium nitroprusside (SNP, 100 µM) was used as NO donor. n = 8. **c** Mitochondrial network continuity calculation using Matlab software. Continuous signal lengths were measured and divided by all object number detected to result in the average length of objects. n = 9–19. Cyanide agent (CCCP, 5 µM) was used to decouple mitochondrial networks. **d** Representative pictures of the mitochondrial network staining using the Mitotracker green labeling. Blue: cell nucleus. Bar: 10 µm. Control groups (C) received only culture medium. On all graphs, values are presented as means ± SEM. Statistical analysis: one-way Anova followed by Bonferroni post-test, *, p < 0.05; ***, p < 0.001

To reveal the reason of the decreased cell viability and barrier integrity in primary rat brain endothelial cells after l-ornithine treatment (20 mM, 24 h) oxidative stress within the cells was investigated. We observed elevated ROS generation within the cells compared to the control group, while no change was seen in case of the NO species (Fig. 7). Since we also noted mitochondrial changes in the TEM study, the mitochondrial network integrity was also visualized using Mitotracker staining. The originally continuous network became fragmented after l-ornithine treatment similarly to the well-known mitochondrial chain uncoupler cyanide agent (Fig. 7c, d). No change was measured in the mitochondrial membrane potential neither after short-term or prolonged treatment with l-ornithine (see Additional file 1: Fig. S2.) Neither did we find any change in the NF-κB nuclear translocation after l-ornithine treatment (see Additional file 1: Fig. S3).

### l-ornithine-induced glycocalyx damage and zeta potential changes in cultured brain endothelial cells

**Fig. 8:**
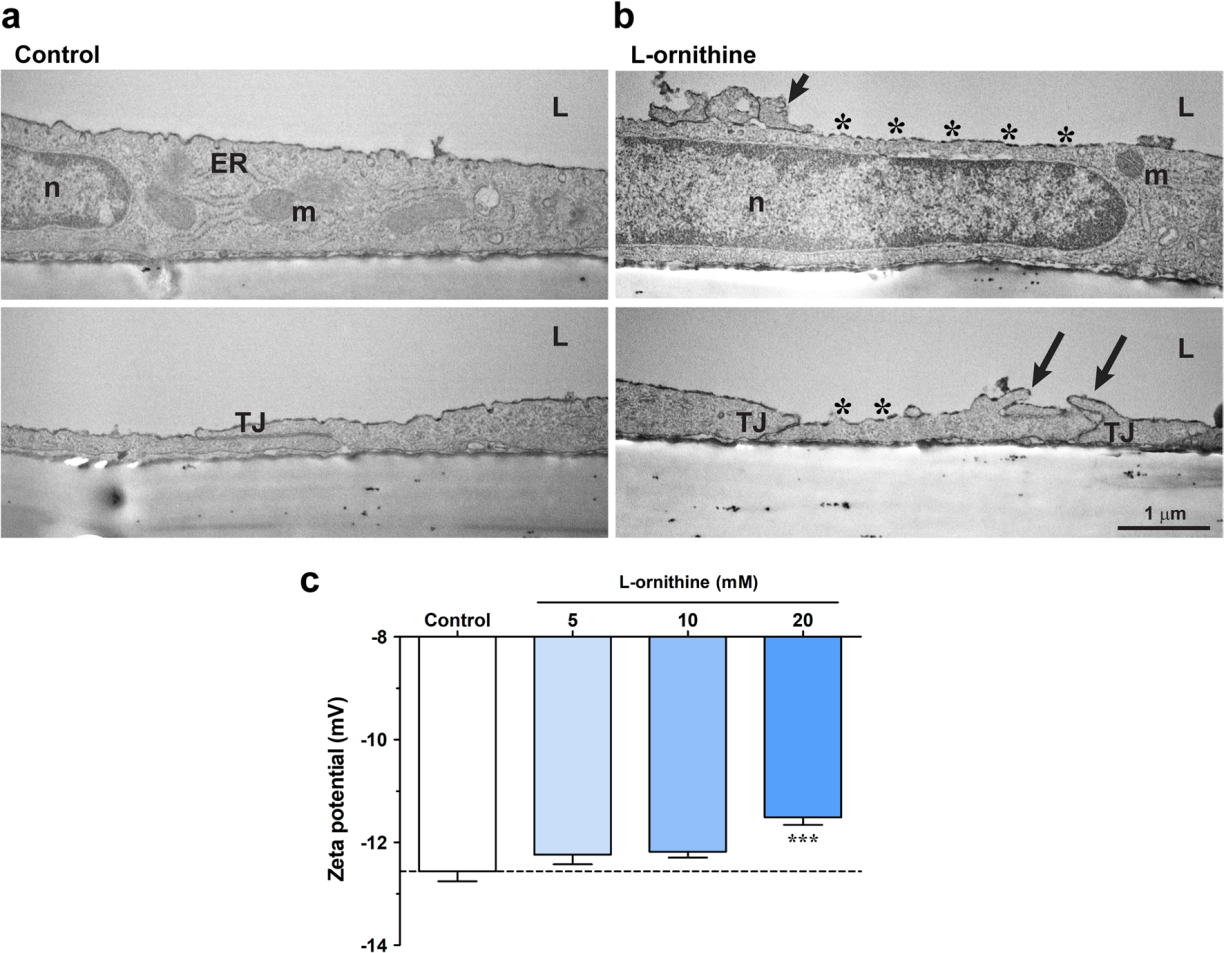
**a** Transmission electron micrographs of primary rat brain endothelial cells kept in a triple BBB co-culture model. Effect of l-ornithine (l-orn, 20 mM, 24 h) treatment was investigated on the ultrastructural changes. The negatively charged surface glycocalyx was labeled with the cationic dye Alcian blue. ER: endoplasmic reticulum, L: luminal side, m: mitochondria, n: nucleus, TJ: intercellular tight junction. Black arrows in the l-ornithine treated group point to plasma membrane deformities. Asterisks show irregular glycocalyx staining by Alcian blue. Bar: 1 µm. **b** Effect of direct l-ornithine (l-orn, 20 mM) treatment on the surface charge of cultured rat primary brain endothelial cells. Values presented are means ± SEM. n = 3–10 with a total of 15–111 rounds of measurements. Statistical analysis: one-way Anova followed by Bonferroni post-test, ***, p < 0.001

Similarly to the in vivo study, we were curious about the ultrastructural changes in primary brain endothelial cells after l-ornithine treatment (20 mM, 24 h). We found major ultrastructural changes compared to the control group: (i) surface glycocalyx discontinuity visualized by Alcian blue, (ii) altered mitochondrial morphology and (iii) increased number of vesicular profiles associated with the plasma membranes (Fig. 8a-b; Table 2). Cultured brain endothelial cells showed a healthy morphology in the control group. The luminal endothelial surface was smooth, with protrusions in about the third of the cases (Table 2), similarly to the in vivo observations (Table 1). In 90% of the analyzed control cells a strong and continuous glycocalyx staining was observed, while the l-ornithine treated group showed in more than 70% of the cases a weak or discontinuous surface glycocalyx pattern (Table 2), corroborating the in vivo findings (Table 1). While TJs and mitochondria were intact in the control cells, junctional discontinuity (22%) and mitochondria with ultrastructure changes (47%), less or disappeared cristae, spherical shape, were detected in the l-ornithine treated group (Table 2). There was only a moderate increase in the vacuolization of l-ornithine treated cells as compared to the control group. We found a pronounced alteration in the number of vesicular profiles at the luminal and abluminal membranes of the cells (Table 2). The average amount of vesicles associated to the plasma membranes in the images was significantly higher in the l-ornithine treated group, especially at the abluminal membrane.

Since we previously found that surface glycocalyx changes are directly linked to the changes of surface zeta potential [36, 37], we investigated the effect of the cationic l-ornithine treatment (5–20 mM) on the negative zeta potential of brain endothelial cells. We found that the effect of l-ornithine was concentration dependent, and the cells’ zeta potential became significantly more positive after the treatment with the highest, 20 mM concentration, suggesting a direct interaction with the cell surface (Fig. 8c).

## Discussion

CNS complications are observed in about one tenth of all severe AP cases [8]. In recent years, more and more clinicians described case studies of posterior reversible encephalopathy syndrome emerging during or after AP [38, 39]. Prompt recognition and treatment can lead to better survival and good prognosis among these patients [40]. Therefore, to understand the role of BBB, as a potential site of damage leading to neurological symptoms is of high clinical importance also during AP.

**Fig. 9:**
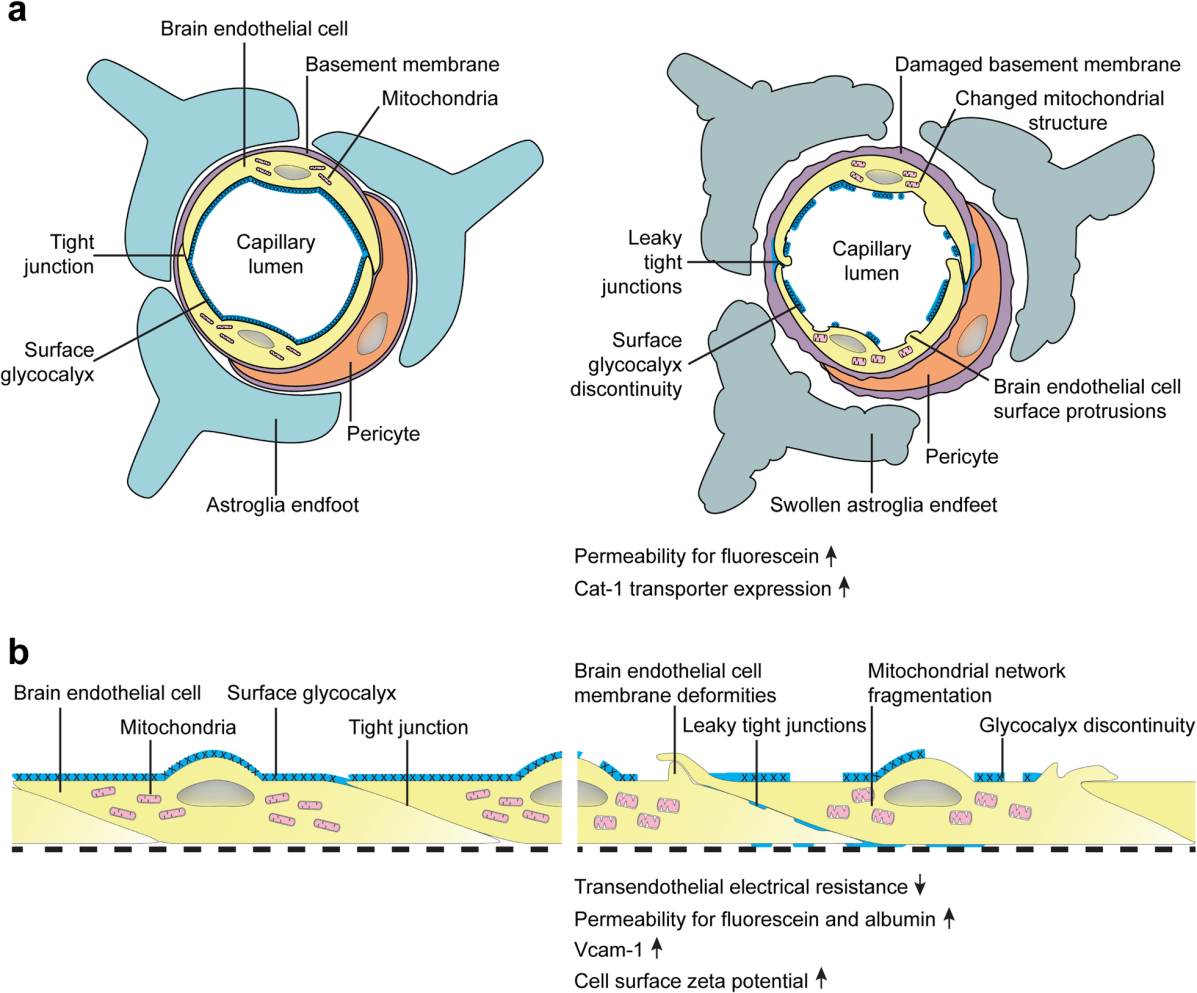
Schematic diagrams of the BBB in vivo (**a**) and brain endothelial cells in vitro (**b**) highlighting the most characteristic changes detected after the induction of AP with l-ornithine (**a**) and after the direct treatment of brain endothelial cells with l-ornithine (**b**)

Here we characterized the permeability and morphological changes in rat microvessels using the l-ornithine-induced non-invasive AP model (Fig. 9). We found that blood vessels in the brain cortex and pancreas of rats with AP were highly permeable for the small molecular weight tracer fluorescein, but not for the larger tracer albumin, pointing to a higher paracellular permeability. No changes in marker molecule extravasation were observed in the liver or the lung. Previously, our laboratory described decreased BBB integrity in the taurocholic acid induced AP rat model for both albumin and fluorescein [12]. Other groups have also observed BBB leakage in animal AP models: albumin flux increase was seen in a sodium choleate infusion model [41], an l-arginine i.p. injection study [42], and another taurocholate model [43]. These corroborate our findings and show that both in the invasive and non-invasive severe rat AP models BBB integrity is compromised.

In the cationic amino acid l-ornithine-induced AP model, vessel leakage was only identified in the brain and the pancreas. Therefore, our aim was to identify, why only these two organs were the most affected in our model, since in other models of AP induced by cerulein or taurocholate, lung vessel [44, 45] and gastrointestinal permeability elevation [46] also occurred. The transport of l-ornithine is exclusively mediated by cationic amino acid transporter family y^+^ in the brain, specifically, the Cat-1 transporter [47]. The system y^+^ has the highest affinity for arginine, followed by lysine and ornithine [47]. It is known that Cat-1 is highly expressed at the BBB both in vivo and in vitro [48–51] and in the human pancreas, but low or no expression is found in the liver or lung [52]. These literature data correlate with our findings about the basal *Cat-1* gene expression (Fig. 2). We found that in the l-ornithine-induced AP model, *Cat-1* gene expression levels were elevated in brain microvessels and in the pancreas, the organs which showed an elevated vessel permeability for fluorescein. No change was found in *Cat-1* expression of the liver and lung, where no extravasation of the marker molecules was measured. Therefore, we hypothesize, that in the current AP model the presence of l-ornithine in the blood and its direct interaction with and Cat-1 mediated uptake into the cells of BBB, brain and pancreas causes a higher sensitivity to permeability elevation and subsequent edema.

To characterize the ultrastructural changes in the brain and the pancreas, TEM was performed on vehicle treated and AP animal groups. Inflammation, organ failure and edema are well-known in AP [5, 6]. Here we also detected by TEM damaged mitochondria, tissue edema and necrosis, as well as severe endothelial injury in the pancreas. These observations are in concordance with previous observations on tissue necrosis, especially in pancreatic acinar cells, along with the presence of apoptotic bodies in the same AP model [14]. Similar observations were made on damaged mitochondria and swollen capillaries in the pancreas of cerulein-induced AP model [53, 54]. Irregularities in endothelial lining in the pancreas capillaries were found in a canine AP model using electron microscopic analysis [55].

Systemic inflammation affects the BBB in various diseases resulting in CNS pathologies [56]. BBB dysfunction and increased permeability have been well described in AP models, yet, ultrastructural changes at the level of brain microvessels in AP have not been studied in detail. In l-ornithine-induced AP we observed changes in mitochondria ultrastructure, vacuolization, damage of the basal lamina and Alcian blue leakage in endothelial cells of brain capillaries. Swollen glial endfeet surrounded the affected microvessels. These morphological changes of microvessels were very similar to those we found in the pancreas of rats in the current l-ornithine-induced AP model. In addition, as a novel approach, we also focused on the changes of the glycocalyx, which is present on the surface of vascular endothelial cells and regulates their functions [57]. This sugar–protein mesh covering the surface of brain capillaries is one of the thickest in the cardiovascular system and forms an important physicochemical barrier at the BBB [23, 58]. In our AP model, we visualized the glycocalyx with the cationic probe Alcian blue, which binds to the highly negatively charged glycocalyx residues and due to its copper content can be visualized by TEM [59]. We observed discontinuity of the endothelial surface glycocalyx staining both in the brain cortex and the pancreas microvessels in the AP group. In addition, in the brain capillaries Alcian blue staining appeared on the abluminal side of the brain endothelial cells in the disease model, indicating a barrier leakage which is in agreement with the observed increase in permeability for fluorescein in the present model.

Our results suggest a more pronounced endothelial damage on the ultrastructural level in the pancreas than in the brain. One of the possible explanation can be that brain capillaries are more robust than microvessels of other organs due to the thicker glycocalyx on the surface of brain capillary endothelium, as it was described in a mouse study [60]. After treatment with bacterial lipopolysaccharide brain microvessels showed the most intact glycocalyx and low permeability for albumin, while in the heart and the lung the endothelial surface glycocalyx disappeared or was much sparser and a higher permeability to albumin was measured [60]. This is in agreement with our observations on the permeability and ultrastructural changes in brain and pancreas microvessels in the present rat AP model. Both our results and the mouse study highlight the glycocalyx as a specific protective mechanism at the BBB in systemic inflammation and pathologies.

Several factors were identified contributing to the pathomechanism of AP and AP models. Elevation of cytokine levels is observed in animal models of AP [12] including the l-ornithine-induced AP model [14] used in our study, as well as in patients at the early phase of AP [61]. Cytokines are well-known potent mediators to increase the permeability of the BBB [62–64]. In addition, in severe AP pancreatic enzymes released to the circulation affect both BBB and brain functions. Elevated levels of serum trypsin activate phospholipase A2, which converts pancreatic lecithins and cephalins to toxic isoforms, which damage the BBB by destroying membrane phospholipids, and facilitate demyelination [65]. A further proof of this hypothesis is that antiphospholipase A2 activity is linked to cerebrovascular protection and attenuation of BBB damage and vasogenic edema formation in animal models of neurological diseases [66, 67]. The trypsin-induced changes, together with elevated blood levels of lipase, elastase and amylase coexacerbate brain edema and CNS dysfunction [68]. In the l-ornithine model, pancreatic edema is visible already after 6 h of the treatment, when trypsin and amylase activity increases, and NF-κB activation and tissue necrosis is detectable [14, 19]. Thus, according to previous observations cytokine release, excessive pancreatic enzyme production and activation of different signal transduction pathways can be identified as possible players contributing to BBB dysfunction and barrier opening in AP animal models and in patients. We hypothesized that besides these mechanisms l-ornithine as a cationic amino acid might exert a direct effect on brain endothelial cell integrity and functions contributing to BBB changes. Therefore, we aimed to reveal the direct effect of l-ornithine treatment on rat brain capillary endothelial cell function and morphology using a primary cell based culture model (Fig. 9).

First, we measured cell viability after direct l-ornithine treatment, which has not yet been studied on primary rat brain endothelial cells. Supposing a uniform tissue distribution, we estimated that with the injection of 3 g/kg l-ornithine, the levels of this amino acid can reach ~ 20 mM concentration in tissues right after injection. We selected the 20 mM concentration based on this in vivo C_max_ estimation. l-ornithine at 20 mM concentration for 24 h caused a mild, but significant decrease in impedance, but it did not change the metabolic activity of the cells. In concordance with our findings, in a cell culture study l-ornithine at 10 mM concentration had no effect on the metabolic activity of retinal pigment epithelial cells [69]. Furthermore, l-ornithine, as an orally administered everyday nutritional additive, has been found effective to reduce stress and fatigue in human volunteers [70, 71].

Using the triple BBB co-culture model, we observed a barrier integrity change after l-ornithine treatment both for the electrical resistance and marker molecule permeability. Decrease in TEER on cultured brain capillary endothelial cells following treatment with lysine and arginine, cationic amino acids, was already reported in the 0.2–200 mM concentration range [72]. Permeability changes have not yet been studied before after l-ornithine treatment on cultured brain endothelial cells. We found that in agreement with our rat experiments, the flux of fluorescein was elevated on the triple BBB co-culture model. In addition, an increase in albumin permeability was also seen on the in vitro model. These findings correspond to the observed TEER decrease. The morphological data also support the functional barrier integrity results. Discontinuity of the immunostaining for claudin-5, occludin, ZO-1 and β-catenin was detected at the intercellular junctions. A decrease in the junctional/cytoplasmic ratio of the fluorescence intensity of ZO-1 and β-catenin indicates the redistribution of these linker proteins from the plasma membrane to the cytoplasm. This change suggests TJ perturbation, as we already observed in our recent study [33] and is in concordance with the central role of ZO-1 and β-catenin as nuclear signaling proteins in barrier regulation in endothelial cells [20, 73]. l-ornithine treatment elevated the expression of Vcam-1 pointing to endothelial cell activation, while no change was seen for Icam-1. These two adhesion molecules are activated during inflammation [74]. However, in atherosclerosis, as well as in TNFα-induced brain injury in murine models, the importance of Vcam-1 activation was much more substantial than that of Icam-1 [75, 76].

It was already described that in the l-ornithine-induced AP animal model beside the cytokine and enzyme level elevations mitochondrial injury, NO and the activation of NF-κB signaling also contribute to the development of AP [14, 16]. Our aim was to test whether l-ornithine has a direct effect on any of these mechanisms in brain endothelial cells. No change in NO levels or NFκB nuclear translocation after l-ornithine treatment was found, therefore, in the in vitro study NO or the activation of the NFκB signaling pathway probably are not the cause of the l-ornithine-induced decrease in brain endothelial cell barrier integrity. On the contrary, l-ornithine caused a significant elevation in ROS production in primary brain endothelial cells. Corresponding to our observations, l-ornithine was found to cause significant ROS generation in pancreatic acinar cells at the same 20 mM concentration [77]. We observed that prolonged treatment with l-ornithine broke up the continuous network of mitochondria, while no change was seen in the mitochondrial membrane potential. Indeed, the ROS increasing effect of 20 mM l-ornithine in acinar cells was blocked through a mitochondria-targeted antioxidant [77] highlighting the central role of ROS and mithochondrial changes in the effect of l-ornithine.

To analyze the ultrastructural changes after l-ornithine treatment, we performed TEM on cultured primary brain endothelial cells for the first time. Glycocalyx is a highly negatively charged protection system at the surface of brain endothelial cells, also present in cell cultures [23]. In this study, we identified discontinued staining of the surface glycocalyx, alterations in mitochondrial ultrastructure and increased number of vesicular profiles associated with the plasma membranes in primary brain endothelial cells, corroborating the TEM findings of the in vivo study. The increased number of vesicles at the abluminal membrane could also explain in the l-ornithine treated BBB culture model the highly elevated albumin permeability, which is associated with adsorptive mediated endocytosis [20]. We observed that the thickness of the glycocalyx on cultured brain endothelial cells, stained by Alcian blue, was thinner and less electron dense than in brain capillaries. Although a direct glycocalyx thickness evaluation of brain capillary microvessels and cultured brain microvascular endothelial cells has not yet been performed in the same study, in peripheral vascular endothelium a comparison of glycocalyx thickness in vivo and in vitro was made [78]. The endothelial cell surface glycocalyx of venules was 20-times thicker than in cultured human umbilical vein or bovine aorta endothelial cells [78]. Our TEM observations are in concordance with these data.

With the measurement of surface zeta potential, we demonstrated, that l-ornithine treatment directly changed the highly negatively charged surface potential of primary brain endothelial cells to more positive. It was described that cationic drug molecules, such as lidocaine or kyotorphin-ibuprofen make the basal negative net surface charge of brain endothelial cells more positive [33, 79]. Based on our measurements and these observations, we propose that l-ornithine directly interacts with the endothelial surface glycocalyx resulting in a surface potential change. Since the negative charge of the glycocalyx is important to keep up healthy ionic homeostasis and provide a barrier for larger charged molecules [80], change in this delicate balance of brain endothelial cells surface charge can result in the loss of adaptation and proneness to BBB damage [81].

## Conclusion

By studying BBB and brain endothelial cell-related changes, we could shed light on the mechanism of brain microvasculature-related damage in the l-ornithine-induced AP model. Our present findings together with literature data suggest a general, AP model independent BBB damage in pancreatitis, that might be important to understand CNS complications in this disease. We revealed BBB and glycocalyx alterations in this rat AP model, for the first time. Our data on l-ornithine-induced changes on a culture model of the BBB suggest a direct interaction of the cationic amino acid l-ornithine with brain endothelial cells. This direct effect can be related to mitochondrial injury, oxidative stress, changes in barrier integrity and BBB morphology. Endothelial surface glycocalyx injury was revealed both in vivo and in vitro, as an additional novel component of the BBB-related pathological changes in AP.

## Supplementary Information

**Additional file 1: Figure S1**. Transmission electron micrographs of the pancreas from rats with acute pancreatitis induced by l-ornithine (n=4). The negatively charged surface glycocalyx was labeled with the cationic dye Alcian blue. AB: Alcian blue extravasation, L: vessel lumen, m: mitochondria, TJ: intercellular tight junction. Stars label perivascular tissue necrosis. # symbols point out edema around microvessels. $ symbols show intracellular edema and disintegration of the ultrastructure indicating severe endothelial injury in the pancreas. Scale bars: 500 nm. **Figure S2**. Mitochondrial membrane potential measurement after a immediate and b 24 h lornithine pre-treatment (lorn; 20 mM) on primary brain endothelial cells. In the pre-treatment experiments data were compared to assay buffer-treated control cells. Data is presented as means ± SEM. CCCP: carbonyl cyanide 3-chlorophenylhydrazone. C: control. Values presented are means ± SEM. n=3. c Representative fluorescent images taken during the live cell imaging. Red: TMRM dye. Scale bar: 20 µm. **Figure S3**. Measurement of the nuclear translocation of NFκB after short term lornithine treatment (20 mM, 1 h) in primary brain endothelial cells. Bacterial lipopolysaccharide (LPS, 1 µg/ml) was used as a reference agent to induce inflammation. C: control group treated with culture medium. a Nucleus/cytoplasm ratio of the NFκB fluorescent staining calculated using ImageJ. Values presented are means ± SEM. Statistical analysis: one-way Anova followed by Bonferroni post-test, ***, p< 0.001. n=2. b Representative fluorescent images of NFκB nuclear translocation. Scale bar: 50 µm. **Table S1**. List of antibodies used in this study.

## Data Availability

The dataset used and/or analysed during the current study are available from the corresponding author on reasonable request.

## Abbreviations

AP: Acute pancreatitis
BBB: Blood–brain barrier
BSA: Bovine serum albumin
Cat-1: Cationic amino acid transporter-1
CCCP: Carbonyl cyanide 3-chlorophenylhydrazone
CNS: Central nervous system
DAF-FM: 4-Amino-5-methylamino-2’,7’-difluorofluorescein diacetate
DCFDA: Chloromethyl-dichloro-dihydro-fluorescein diacetate
EBA: Evans blue-labelled albumin
Icam-1: Intercellular adhesion molecule-1
MTT: 3-(4,5-Dimethylthiazol-2-yl)-2,5-diphenyltetrazolium bromide
NO: Nitric oxide
H_2_O_2_: Hydrogen peroxide
P_e_: Permeability coefficient for endothelial monolayers
PBS: Phosphate buffered saline
PFA: Paraformaldehyde
ROS: Reactive oxygen species
SF: Sodium fluorescein
SNP: Sodium nitroprusside
TEER: Transendothelial electrical resistance
TEM: Transmission electron microscopy
TJ: Tight junction
Vcam-1: Vascular cell adhesion molecule-1
ZO-1: Zonula occludens protein-1

## References

[1] Carnovale A, Rabitti PG, Manes G, Esposito P, Pacelli L (2005). Mortality in acute pancreatitis: is it an early or a late event?. JOP.

[2] Shah AP, Mourad MM, Bramhall SR (2018). Acute pancreatitis: current perspectives on diagnosis and management. J Inflamm Res.

[3] Mederos MA, Reber HA, Girgis MD (2021). Acute pancreatitis: a review. JAMA.

[4] Pitchumoni CS, Patel NM, Shah P (2005). Factors influencing mortality in acute pancreatitis: can we alter them?. J Clin Gastroenterol.

[5] Sah RP, Garg P, Saluja AK (2012). Pathogenic mechanisms of acute pancreatitis. Curr Opin Gastroenterol.

[6] Banks PA, Bollen TL, Dervenis C, Gooszen HG, Johnson CD (2013). Classification of acute pancreatitis–2012: revision of the Atlanta classification and definitions by international consensus. Gut.

[7] Ohkubo T, Shiojiri T, Matsunaga T (2004). Severe diffuse white matter lesions in a patient with pancreatic encephalopathy. J Neurol.

[8] Párniczky A, Kui B, Szentesi A, Balázs A, Szűcs Á (2016). Prospective, multicentre, nationwide clinical data from 600 cases of acute pancreatitis. PLoS ONE.

[9] Hágendorn R, Vincze Á, Izbéki F, Gajdán L, Gódi S (2020). Development of disturbance of consciousness is associated with increased severity in acute pancreatitis. Pancreatology.

[10] Yang X, Yao L, Fu X, Mukherjee R, Xia Q (2020). Experimental acute pancreatitis models: history, current status, and role in translational research. Front Physiol.

[11] Aho HJ, Koskensalo SM, Nevalainen TJ (1980). Experimental pancreatitis in the rat. Sodium taurocholate-induced acute haemorrhagic pancreatitis. Scand J Gastroenterol.

[12] Farkas G, Márton J, Nagy Z, Mándi Y, Takács T (1998). Experimental acute pancreatitis results in increased blood-brain barrier permeability in the rat: a potential role for tumor necrosis factor and interleukin 6. Neurosci Lett.

[13] Mizunuma T, Kawamura S, Kishino Y (1984). Effects of injecting excess arginine on rat pancreas. J Nutr.

[14] Rakonczay Z, Hegyi P, Dósa S, Iványi B, Jármay K (2008). A new severe acute necrotizing pancreatitis model induced by L-ornithine in rats. Crit Care Med.

[15] Biczó G, Hegyi P, Dósa S, Balla Z, Venglovecz V (2011). Aliphatic, but not imidazole, basic amino acids cause severe acute necrotizing pancreatitis in rats. Pancreas.

[16] Rakonczay Z, Jármay K, Kaszaki J, Mándi Y, Duda E (2003). NF-kappaB activation is detrimental in arginine-induced acute pancreatitis. Free Radic Biol Med.

[17] Biczó G, Hegyi P, Sinervirta R, Berczi S, Dósa S (2010). Characterization of polyamine homeostasis in l-ornithine-induced acute pancreatitis in rats. Pancreas.

[18] Biczo G, Vegh ET, Shalbueva N, Mareninova OA, Elperin J (2018). Mitochondrial dysfunction, through impaired autophagy, leads to endoplasmic reticulum stress, deregulated lipid metabolism, and pancreatitis in animal models. Gastroenterology.

[19] Kui B, Balla Z, Végh ET, Pallagi P, Venglovecz V (2014). Recent advances in the investigation of pancreatic inflammation induced by large doses of basic amino acids in rodents. Lab Invest.

[20] Abbott NJ, Patabendige AA, Dolman DE, Yusof SR, Begley DJ (2010). Structure and function of the blood-brain barrier. Neurobiol Dis.

[21] Abbott NJ, Friedman A. Overview and introduction: the blood-brain barrier in health and disease. Epilepsia. 2012; 53(Suppl 6(0 6):1–6. doi: 10.1111/j.1528-1167.2012.03696.x. 10.1111/j.1528-1167.2012.03696.x PMC3625728 23134489

[22] Profaci CP, Munji RN, Pulido RS, Daneman R (2020). The blood-brain barrier in health and disease: important unanswered questions. J Exp Med.

[23] Walter FR, Santa-Maria AR, Mészáros M, Veszelka S, Dér A (2021). Surface charge, glycocalyx, and blood-brain barrier function. Tissue Barriers.

[24] Stokes WS (2002). Humane endpoints for laboratory animals used in regulatory testing. ILAR J.

[25] Kui B, Balla Z, Vasas B, Végh ET, Pallagi P, Kormányos ES (2015). New insights into the methodology of L-arginine-induced acute pancreatitis. PLoS ONE.

[26] Deli MA, Németh L, Falus A, Abrahám CS (2000). Effects of N, N-diethyl-2-[4-(phenylmethyl)phenoxy]ethanamine on the blood-brain barrier permeability in the rat. Eur J Pharmacol.

[27] Walter FR, Veszelka S, Pásztói M, Péterfi ZA, Tóth A (2015). Tesmilifene modifies brain endothelial functions and opens the blood-brain/blood-glioma barrier. J Neurochem.

[28] Szczepkowska A, Harazin A, Barna L, Deli MA, Skipor J (2021). Identification of reference genes for circadian studies on brain microvessels and choroid plexus samples isolated from rats. Biomolecules.

[29] Nakagawa S, Deli MA, Kawaguchi H, Shimizudani T, Shimono T (2009). A new blood-brain barrier model using primary rat brain endothelial cells, pericytes and astrocytes. Neurochem Int.

[30] Barna L, Walter FR, Harazin A, Bocsik A, Kincses A (2020). Simvastatin, edaravone and dexamethasone protect against kainate-induced brain endothelial cell damage. Fluids Barriers CNS.

[31] Walter FR, Gilpin TE, Herbath M, Deli MA, Sandor M (2020). A novel in vitro mouse model to study mycobacterium tuberculosis dissemination across brain vessels: a combination granuloma and blood-brain barrier mouse model. Curr Protoc Immunol.

[32] Deli MA, Abrahám CS, Kataoka Y, Niwa M (2005). Permeability studies on in vitro blood-brain barrier models: physiology, pathology, and pharmacology. Cell Mol Neurobiol.

[33] Santa-Maria AR, Walter FR, Valkai S, Brás AR, Mészáros M (2019). Lidocaine turns the surface charge of biological membranes more positive and changes the permeability of blood-brain barrier culture models. Biochim Biophys Acta Biomembr.

[34] Hülper P, Veszelka S, Walter FR, Wolburg H, Fallier-Becker P (2013). Acute effects of short-chain alkylglycerols on blood-brain barrier properties of cultured brain endothelial cells. Br J Pharmacol.

[35] Lénárt N, Walter FR, Bocsik A, Sántha P, Tóth ME (2015). Cultured cells of the blood-brain barrier from apolipoprotein B-100 transgenic mice: effects of oxidized low-density lipoprotein treatment. Fluids Barriers CNS.

[36] Kincses A, Santa-Maria AR, Walter FR, Dér L, Horányi N (2020). A chip device to determine surface charge properties of confluent cell monolayers by measuring streaming potential. Lab Chip.

[37] Kanyo N, Kovacs KD, Saftics A, Szekacs I, Peter B (2020). Glycocalyx regulates the strength and kinetics of cancer cell adhesion revealed by biophysical models based on high resolution label-free optical data. Sci Rep.

[38] Murphy T, Al-Sharief K, Sethi V, Ranger GS (2015). Posterior reversible encephalopathy syndrome (PRES) after acute pancreatitis. West J Emerg Med.

[39] Bonavia LJ, Jackson J, Jurevics R (2020). Posterior reversible encephalopathy syndrome in acute pancreatitis: a rare stroke mimic. BMJ Case Rep.

[40] Hobson EV, Craven I, Blank SC (2012). Posterior reversible encephalopathy syndrome: a truly treatable neurologic illness. Perit Dial Int.

[41] Ding Z, Liu J, Lin R, Hou XH (2012). Experimental pancreatitis results in increased blood-brain barrier permeability in rats: a potential role of MCP-1. J Dig Dis.

[42] Ou X, Hua Y, Liao X, Gong C, Kang Y (2018). Cognitive impairments induced by severe acute pancreatitis are attenuated by berberine treatment in rats. Mol Med Rep.

[43] Lin R, Chen F, Wen S, Teng T, Pan Y (2018). Interleukin-10 attenuates impairment of the blood-brain barrier in a severe acute pancreatitis rat model. J Inflamm (Lond).

[44] Bellows CF, Brain JD (2003). Role of fibronectin in pancreatitis-associated lung injury. Dig Dis Sci.

[45] Meng Y, Zhang M, Xu J, Liu XM, Ma QY (2005). Effect of resveratrol on microcirculation disorder and lung injury following severe acute pancreatitis in rats. World J Gastroenterol.

[46] Ryan CM, Schmidt J, Lewandrowski K (1993). Gut macromolecular permeability in pancreatitis correlates with severity of disease in rats. Gastroenterology.

[47] O'Kane RL, Viña JR, Simpson I, Zaragozá R, Mokashi A (2006). Cationic amino acid transport across the blood-brain barrier is mediated exclusively by system y+. Am J Physiol Endocrinol Metab.

[48] Enerson BE, Drewes LR (2006). The rat blood-brain barrier transcriptome. J Cereb Blood Flow Metab.

[49] Vanlandewijck M, He L, Mäe MA, Andrae J, Ando K (2018). A molecular atlas of cell types and zonation in the brain vasculature. Nature.

[50] Veszelka S, Tóth A, Walter FR, Tóth AE, Gróf I (2018). Comparison of a rat primary cell-based blood-brain barrier model with epithelial and brain endothelial cell lines: gene expression and drug transport. Front Mol Neurosci.

[51] Yang AC, Vest ET, Kern F, Lee DP, Maat CA, et al. A human brain vascular atlas reveals diverse cell mediators of Alzheimer’s disease risk. bioRxiv 2021.04.26.441262; doi: 10.1101/2021.04.26.441262 10.1038/s41586-021-04369-3 PMC9635042 35165441

[52] Uhlén M, Fagerberg L, Hallström BM, Lindskog C, Oksvold P (2015). Proteomics. Tissue-based map of the human proteome. Science.

[53] Takano S, Kimura T, Kawabuchi M, Yamaguchi H, Kinjo M (1994). Ultrastructural study of the effects of stress on the pancreas in rats. Pancreas.

[54] Andrzejewska A, Dlugosz JW, Augustynowicz A (2005). Effect of endothelin-1 receptor antagonists on histological and ultrastructural changes in the pancreas and trypsinogen activation in the early course of caerulein-induced acute pancreatitis in rats. World J Gastroenterol.

[55] McEntee G, Leahy A, Cottell D, Dervan P, McGeeney K (1989). Three-dimensional morphological study of the pancreatic microvasculature in caerulein-induced experimental pancreatitis. Br J Surg.

[56] Varatharaj A, Galea I (2017). The blood-brain barrier in systemic inflammation. Brain Behav Immun.

[57] Reitsma S, Slaaf DW, Vink H, van Zandvoort MA, Oude Egbrink MG (2007). The endothelial glycocalyx: composition, functions, and visualization. Pflugers Arch.

[58] Reed MJ, Damodarasamy M, Banks WA (2019). The extracellular matrix of the blood-brain barrier: structural and functional roles in health, aging, and Alzheimer’s disease. Tissue Barriers.

[59] Hempel C, Hyttel P, Kurtzhals JA (2014). Endothelial glycocalyx on brain endothelial cells is lost in experimental cerebral malaria. J Cereb Blood Flow Metab.

[60] Ando Y, Okada H, Takemura G, Suzuki K, Takada C (2018). Brain-specific ultrastructure of capillary endothelial glycocalyx and its possible contribution for blood brain barrier. Sci Rep.

[61] Baek HS, Lee SJ (2015). A case of posterior reversible encephalopathy syndrome associated with acute pancreatitis and chronic alcoholism. Gen Hosp Psychiatry.

[62] Deli MA, Descamps L, Dehouck MP, Cecchelli R, Joó F (1995). Exposure of tumor necrosis factor-alpha to luminal membrane of bovine brain capillary endothelial cells cocultured with astrocytes induces a delayed increase of permeability and cytoplasmic stress fiber formation of actin. J Neurosci Res.

[63] Abraham CS, Deli MA, Joo F, Megyeri P, Torpier G (1996). Intracarotid tumor necrosis factor-alpha administration increases the blood-brain barrier permeability in cerebral cortex of the newborn pig: quantitative aspects of double-labelling studies and confocal laser scanning analysis. Neurosci Lett.

[64] Harazin A, Bocsik A, Barna L, Kincses A, Váradi J (2018). Protection of cultured brain endothelial cells from cytokine-induced damage by α-melanocyte stimulating hormone. PeerJ.

[65] Gill D, Sheikh N, Shah A, Ruiz VG, Savici D (2017). Diffuse cerebral edema from acute pancreatitis induced by hypertriglyceridemia. Am J Med.

[66] Sztriha L, Joó F, Szerdahelyi P, Eck E, Koltai M (1986). Effects of dexamethasone on brain edema induced by kainic acid seizures. Neuroscience.

[67] Hoda MN, Singh I, Singh AK, Khan M (2009). Reduction of lipoxidative load by secretory phospholipase A2 inhibition protects against neurovascular injury following experimental stroke in rat. J Neuroinflammation.

[68] Zhang XP, Tian H (2007). Pathogenesis of pancreatic encephalopathy in severe acute pancreatitis. Hepatobiliary Pancreat Dis Int.

[69] Nakauchi T, Ando A, Ueda-Yamada M, Yamazaki Y, Uyama M (2003). Prevention of ornithine cytotoxicity by nonpolar side chain amino acids in retinal pigment epithelial cells. Invest Ophthalmol Vis Sci.

[70] Sugino T, Shirai T, Kajimoto Y, Kajimoto O (2008). L-ornithine supplementation attenuates physical fatigue in healthy volunteers by modulating lipid and amino acid metabolism. Nutr Res.

[71] Miyake M, Kirisako T, Kokubo T, Miura Y, Morishita K (2014). Randomised controlled trial of the effects of L-ornithine on stress markers and sleep quality in healthy workers. Nutr J.

[72] Erben M, Decker S, Franke H, Galla HJ (1995). Electrical resistance measurements on cerebral capillary endothelial cells–a new technique to study small surface areas. J Biochem Biophys Methods.

[73] Tornavaca O, Chia M, Dufton N, Almagro LO, Conway DE (2015). ZO-1 controls endothelial adherens junctions, cell-cell tension, angiogenesis, and barrier formation. J Cell Biol.

[74] Hua S (2013). Targeting sites of inflammation: intercellular adhesion molecule-1 as a target for novel inflammatory therapies. Front Pharmacol.

[75] Cybulsky MI, Iiyama K, Li H, Zhu S, Chen M (2001). A major role for VCAM-1, but not ICAM-1, in early atherosclerosis. J Clin Invest.

[76] Marcos-Contreras OA, Greineder CF, Kiseleva RY, Parhiz H, Walsh LR (2020). Selective targeting of nanomedicine to inflamed cerebral vasculature to enhance the blood-brain barrier. Proc Natl Acad Sci U S A.

[77] Chvanov M, Huang W, Jin T, Wen L, Armstrong J (2015). Novel lipophilic probe for detecting near-membrane reactive oxygen species responses and its application for studies of pancreatic acinar cells: effects of pyocyanin and L-ornithine. Antioxid Redox Signal.

[78] Potter DR, Damiano ER (2008). The hydrodynamically relevant endothelial cell glycocalyx observed in vivo is absent in vitro. Circ Res.

[79] Ribeiro MMB, Pinto ART, Domingues MM, Serrano I, Heras M (2011). Chemical conjugation of the neuropeptide kyotorphin and ibuprofen enhances brain targeting and analgesia. Mol Pharm.

[80] Kutuzov N, Flyvbjerg H, Lauritzen M (2018). Contributions of the glycocalyx, endothelium, and extravascular compartment to the blood-brain barrier. Proc Natl Acad Sci U S A.

[81] Ingber DE (2003). Mechanobiology and diseases of mechanotransduction. Ann Med.

