# Supplementary material for: Blood–brain barrier dysfunction in l-ornithine induced acute pancreatitis in rats and the direct effect of l-ornithine on cultured brain endothelial cells
Source: Fluids Barriers CNS. 2022 Feb 17;19:16. doi: 10.1186/s12987-022-00308-0 (PMC8851707; doi:10.1186/s12987-022-00308-0)
Supplement: Supplementary file 1 — Additional file 1: Figure S1. Transmission electron micrographs of the pancreas from rats with acute pancreatitis induced by l-ornithine (n=4). The negatively charged surface glycocalyx was labeled with the cationic dye Alcian blue. AB: Alcian blue extravasation, L: vessel lumen, m: mitochondria, TJ: intercellular tight junction. Stars label perivascular tissue necrosis. # symbols point out edema around microvessels. $ symbols show intracellular edema and disintegration of the ultrastructure indicating severe endothelial injury in the pancreas. Scale bars: 500 nm. Figure S2. Mitochondrial membrane potential measurement after a immediate and b 24 h lornithine pre-treatment (lorn; 20 mM) on primary brain endothelial cells. In the pre-treatment experiments data were compared to assay buffer-treated control cells. Data is presented as means ± SEM. CCCP: carbonyl cyanide 3-chlorophenylhydrazone. C: control. Values presented are means ± SEM. n=3. c Representative fluorescent images taken during the live cell imaging. Red: TMRM dye. Scale bar: 20 µm. Figure S3. Measurement of the nuclear translocation of NFκB after short term lornithine treatment (20 mM, 1 h) in primary brain endothelial cells. Bacterial lipopolysaccharide (LPS, 1 µg/ml) was used as a reference agent to induce inflammation. C: control group treated with culture medium. a Nucleus/cytoplasm ratio of the NFκB fluorescent staining calculated using ImageJ. Values presented are means ± SEM. Statistical analysis: one-way Anova followed by Bonferroni post-test, ***, p< 0.001. n=2. b Representative fluorescent images of NFκB nuclear translocation. Scale bar: 50 µm. Table S1. List of antibodies used in this study. [file 12987_2022_308_MOESM1_ESM.docx]

**Additional file 1**

**Blood-brain barrier dysfunction in l-ornithine induced acute pancreatitis in rats and the direct effect of l-ornithine on cultured brain endothelial cells**

Fruzsina R Walter, András Harazin, Andrea E. Tóth, Szilvia Veszelka, Ana R. Santa-Maria, Lilla Barna, András Kincses, György Biczó, Zsolt Balla, Balázs Kui, József Maléth, László Cervenak, Vilmos Tubak, Ágnes Kittel, Zoltán Rakonczay Jr, Mária A. Deli

**Materials and methods**

**Mitochondrial membrane potential measurement**

Along with the morphological analysis of the mitochondrial network, we were also curious whether the function of mitochondria change. Therefore, we planned a mitochondrial membrane potential measurement, which was performed under constant perfusion of the treatment solutions by real time live cell imaging using an Olympus FV10i-W confocal laser scanning microscope (Olympus, Tokyo, Japan). Cells were cultured on a glass bottom Petri dish (MatTek Corporation, Ashland, MA, USA) coated with collagen type IV (100 µg/ml), and were treated with l‑ornithine either directly (10-40 mM) or after a 24 h pre-treatment (20 mM). Before the start of the experiment cells were loaded with the TMRM dye (100 nM, Thermo Fisher Scientific Inc., Waltham, MA, USA) in Ringer-Hepes for 30 min at 37 °C, then live imaging was started. In the direct treatment setup cells were continuously and gently perfused with the solutions, with a washing step in between with Ringer-Hepes buffer. In the pre-treatment setup baseline mitochondrial membrane potential was recorded and two conditions, culture medium (control) and l‑ornithine treatments were compared. At the end of both experiments cells received a mitochondrial respiratory chain uncoupler cyanide (CCCP, 100 µM) to induce mitochondrial membrane potential changes (positive control). Image analysis was performed with the Olympus FV10i software.

**NFκB nuclear translocation**

To discover, whether the previously observed molecular changes during in vivo l-ornithine treatment (Rakonczay et al, 2008) also play a role in the effects seen on cultured primary brain endothelial cells we performed NFκB staining. After a short term, 60 min l-ornithine treatment, cells were fixed for 10 min at room temperature with ice-cold acetone-methanol solution (1:1 v/v). Cells were immediately rehydrated after fixation with 1% fetal bovine serum in phosphate buffered saline (PBS). Immunohistochemistry and analysis was performed exactly as previously described in our publication (Walter et al, 2015). For this assay we used rabbit anti-NFκB (sc 372, 1 µg/mL, Santa Cruz Biotechnology Inc., Santa Cruz, CA) primary antibody with a goat anti-rabbit secondary antibody (A-11011, 1 µg/mL, ThermoFisher Scientific Inc., Waltham, MA).

**Results**

**Severe edema, tissue necrosis and endothelial damage occur at the ultrastructural level in the pancreas microvessels and acinar cells during acute pancreatitis in rats**


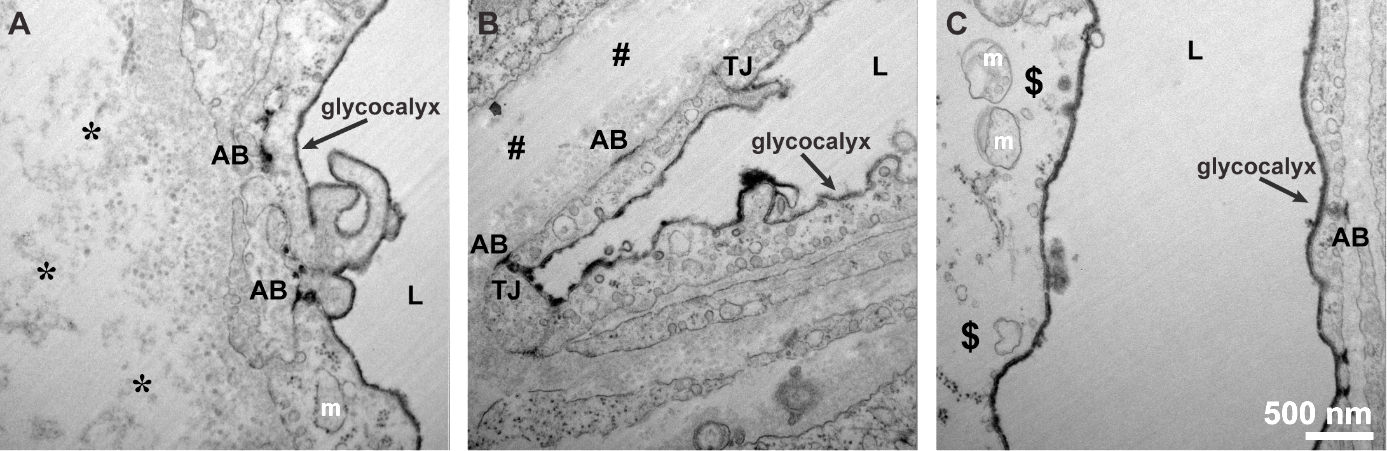


**Figure S1.** Transmission electron micrographs of the pancreas from rats with acute pancreatitis induced by l-ornithine (n=4). The negatively charged surface glycocalyx was labeled with the cationic dye Alcian blue. AB: Alcian blue extravasation, L: vessel lumen, m: mitochondria, TJ: intercellular tight junction. Stars label perivascular tissue necrosis. # symbols point out edema around microvessels. $ symbols show intracellular edema and disintegration of the ultrastructure indicating severe endothelial injury in the pancreas. Scale bars: 500 nm.

**l‑ornithine treatment does not change mitochondrial membrane potential**

Since disorganization of mitochondrial network was observed in cells after 24 h l‑ornithine treatment, we also investigated the effect of l‑ornithine on the mitochondrial membrane potential of primary brain endothelial cells. We observed that neither immediate treatment, nor pre-treatment (24 h) changed the mitochondrial membrane potential visualized by the TMRM dye (Fig. S1). The mitochondrial respiratory chain uncoupler cyanide CCCP decreased the membrane potential immediately along with a sharp fluorescent signal drop (Fig. S1).


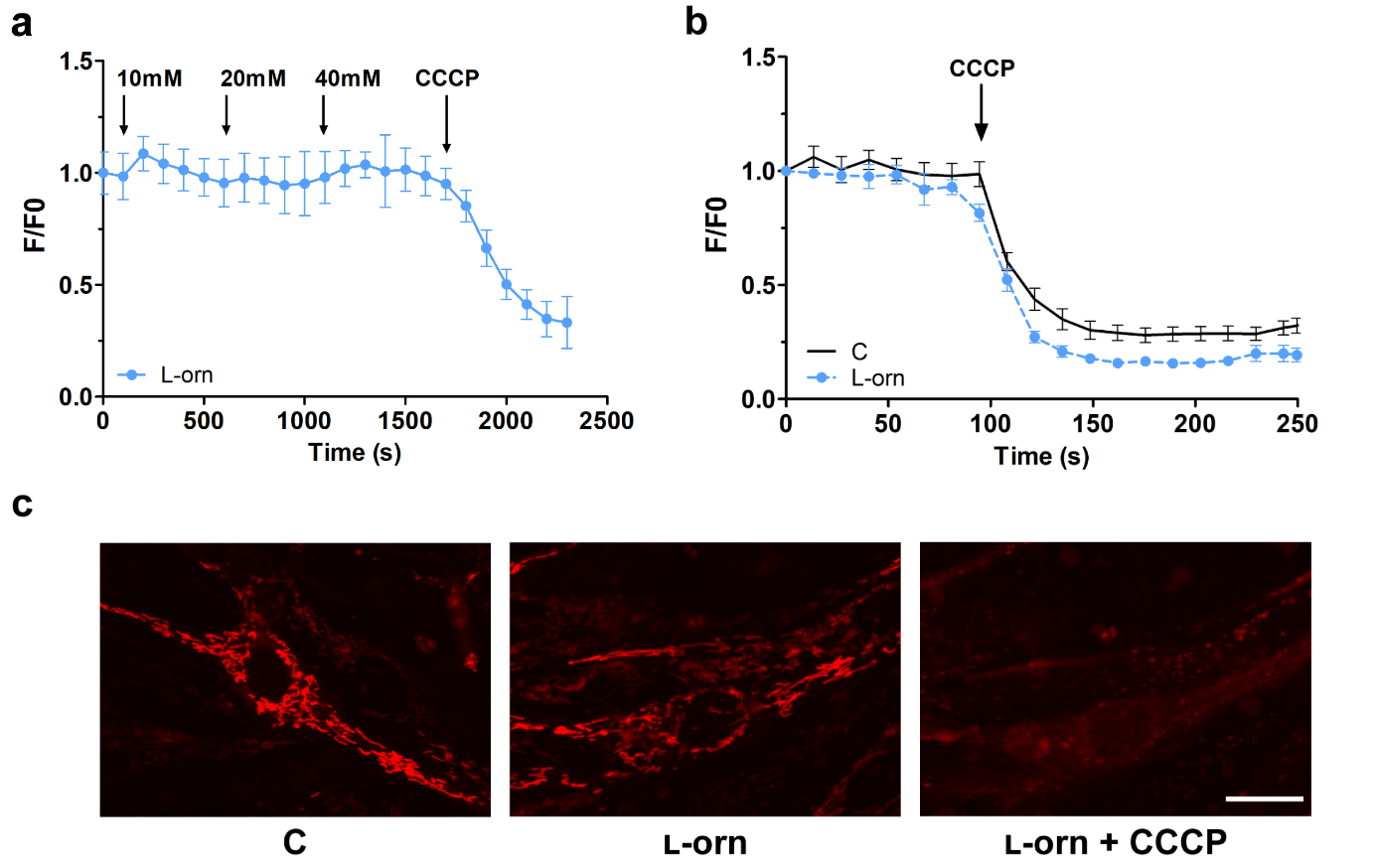


**Figure S2.** Mitochondrial membrane potential measurement after **a** immediate and **b** 24 h l‑ornithine pre-treatment (l‑orn; 20 mM) on primary brain endothelial cells. In the pre-treatment experiments data were compared to assay buffer-treated control cells. Data is presented as means ± SEM. CCCP: carbonyl cyanide 3-chlorophenylhydrazone. C: control. Values presented are means ± SEM. n=3. **c** Representative fluorescent images taken during the live cell imaging. Red: TMRM dye. Scale bar: 20 µm.

**Short-term l‑ornithine treatment does not change NFκB nuclear translocation**

In order to analyze the intracellular mechanisms following short-term (1 h) l‑ornithine treatment we performed immunocytochemical staining for NFκB to identify potential quick nuclear translocation in primary brain endothelial cells. The presence of NFκB in the nucleus was measured by the nucleus/cytoplasm ratio using the immunfluorescent images. l‑ornithine treatment did not elevate the nucleus/cytoplasm ratio of NFκB signal similarly to the control group. Lipopolysaccharide treatment (1 µg/ml, 1 h) elevated this ratio causing a quick translocation (Figure S2).


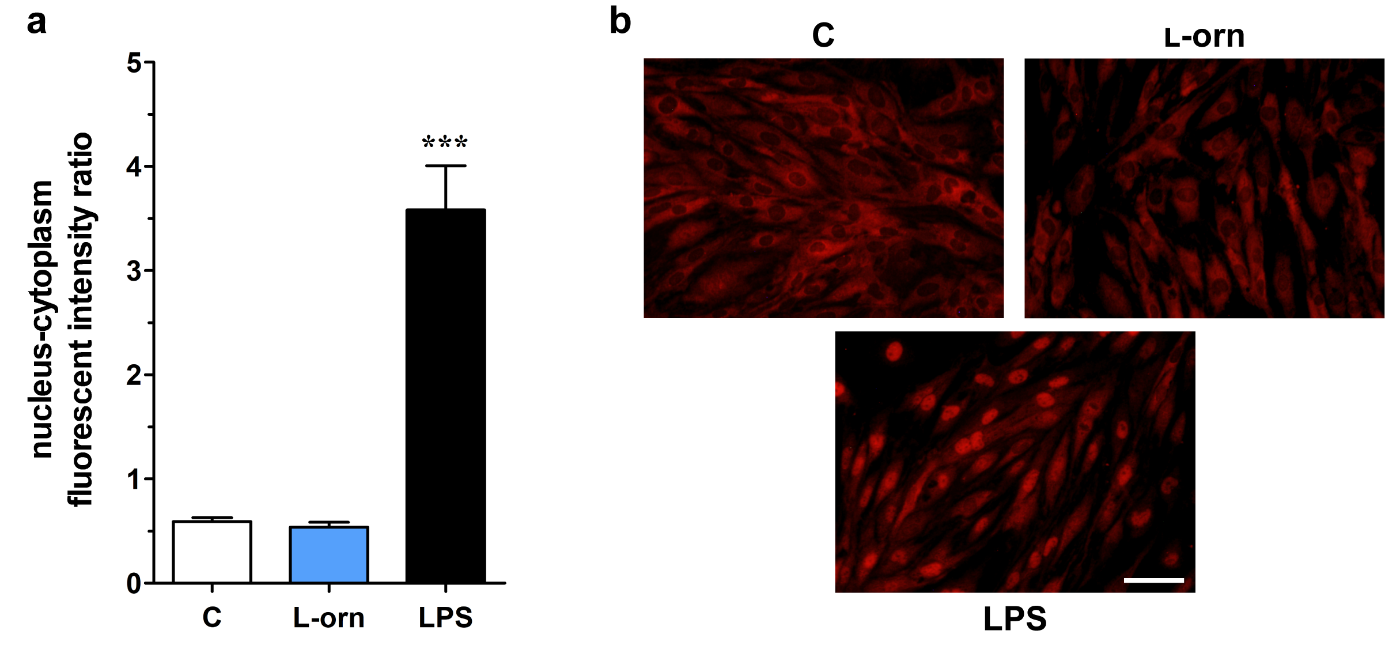


**Figure S3.** Measurement of the nuclear translocation of NFκB after short term l‑ornithine treatment (20 mM, 1 h) in primary brain endothelial cells. Bacterial lipopolysaccharide (LPS, 1 µg/ml) was used as a reference agent to induce inflammation. C: control group treated with culture medium. **a** Nucleus/cytoplasm ratio of the NFκB fluorescent staining calculated using ImageJ. Values presented are means ± SEM. Statistical analysis: one-way Anova followed by Bonferroni post-test, ***, p< 0.001. n=2. **b** Representative fluorescent images of NFκB nuclear translocation. Scale bar: 50 µm.

**Table S1.** List of antibodies used in this study.

| **Antibody name** | **Vendor** | **Catalogue number** | **Host organism** | **Concentrations** | **Antibody registry ID** |  |
| --- | --- | --- | --- | --- | --- | --- |
| **Primary antibodies** | | | | | | |
| Claudin-5 | Sigma Aldrich/Merck | SAB4502981 | rabbit | 1.6 µg/ml | [AB_10753223](http://antibodyregistry.org/AB_10753223) |  |
| Occludin | Thermo Fisher Scientific | 71-1500 | rabbit | 1.25 µg/ml | [AB_2533977](http://antibodyregistry.org/AB_2533977) |  |
| Zonula occludens-1 | Thermo Fisher Scientific | 61-7300 | rabbit | 0.625 µg/ml | [AB_2533938](http://antibodyregistry.org/AB_2533938) |  |
| β-catenin | Sigma Aldrich/Merck | C2206 | rabbit | 1.6 µg/ml | [AB_476831](http://antibodyregistry.org/AB_476831) |  |
| Icam-1 | Thermo Fisher Scientific | MA5407 | mouse | 2 µg/ml | [AB_223596](http://antibodyregistry.org/AB_223596) |  |
| Vcam-1 | Thermo Fisher Scientific | 13-1060-82 | mouse | 2 µg/ml | [AB_529468](http://antibodyregistry.org/AB_529468) |  |
| NFκB | Santa Cruz Biotechnology | sc 372 | rabbit | 1 µg/ml | [AB_632037](http://antibodyregistry.org/AB_632037) |  |
| **Secondary antibodies** | | | | | | |
| Anti-rabbit-CY3 | Sigma Aldrich/Merck | C2306-1ML | sheep | 2.5 µg/ml | [AB_258792](http://antibodyregistry.org/AB_258792) |  |
| Anti-mouse-A488 | Thermo Fisher Scientific | A-11029 | goat | 4 µg/ml | [AB_138404](http://antibodyregistry.org/AB_138404) |  |
| Anti-rabbit A568 | Thermo Fisher Scientific | A-11011 | goat | 1 µg/ml | [AB_143157](http://antibodyregistry.org/AB_143157) |  |
